# PsychoAge and SubjAge: development of deep markers of psychological and subjective age using artificial intelligence

**DOI:** 10.18632/aging.202344

**Published:** 2020-12-08

**Authors:** Alex Zhavoronkov, Kirill Kochetov, Peter Diamandis, Maria Mitina

**Affiliations:** 1 Deep Longevity, Inc, Three Exchange Square, The Landmark, Hong Kong, China; 2 Insilico Medicine, Hong Kong Science and Technology Park (HKSTP), Hong Kong, China; 3 The Buck Institute for Research on Aging, Novato, CA 94945, USA; 4 Singularity University, Mountain View, CA 94040, USA

**Keywords:** psychology of aging, subjective age, deep learning, artificial intelligence, aging clock

## Abstract

Aging clocks that accurately predict human age based on various biodata types are among the most important recent advances in biogerontology. Since 2016 multiple deep learning solutions have been created to interpret facial photos, omics data, and clinical blood parameters in the context of aging. Some of them have been patented to be used in commercial settings. However, psychological changes occurring throughout the human lifespan have been overlooked in the field of “deep aging clocks”.

In this paper, we present two deep learning predictors trained on social and behavioral data from Midlife in the United States (MIDUS) study: (a) PsychoAge, which predicts chronological age, and (b) SubjAge, which describes personal aging rate perception. Using 50 distinct features from the MIDUS dataset these models have achieved a mean absolute error of 6.7 years for chronological age and 7.3 years for subjective age. We also show that both PsychoAge and SubjAge are predictive of all-cause mortality risk, with SubjAge being a more significant risk factor.

Both clocks contain actionable features that can be modified using social and behavioral interventions, which enables a variety of aging-related psychology experiment designs. The features used in these clocks are interpretable by human experts and may prove to be useful in shifting personal perception of aging towards a mindset that promotes productive and healthy behaviors.

## INTRODUCTION

Aging in humans is a process that affects all levels of biological organization: from molecular to systemic. Lower level facets of aging have been studied the most extensively, which has led to the identification of numerous molecular to cellular “hallmarks of aging”: telomere attrition, genome instability, epigenetic dysregulation, shifts in gene expression patterns and metabolic profiles [1–3]. Longitudinal studies of these processes have produced multiple “aging biomarkers”, which are the most convenient and reliable features to determine the extent of the aging-related changes in the human body. The degree of such changes is usually expressed as “biological age” — a numeric value describing how typical the observed biomarker configuration is for healthy chronological age peers within a population [4]. Higher biological age values indicate the higher intensity of aging-related detrimental processes, while lower biological age — higher resilience to them. Traditionally, biological age metrics are designed to resemble the chronological age distribution within a cohort of healthy individuals, while being more predictive of a person’s health status than chronological age itself [5].

Although this approach has yielded impressive results when applied to the study of low-level mechanisms of aging, the results of applying it to the study of high-level, emergent properties of the human organism have remained extremely limited. More specifically, the processes regulating psychological aging and the progression of subjective aging perception are severely understudied. This work focuses primarily on the application of biogerontological methods to the study of the human psyche in hopes to close the gap in our understanding of organismal aging.

The perception of one’s own mortality, physical frailty, and the inexorable march of time give birth to the concept of “subjective age”. Most typically measured as the answer to “How old do you feel to be?” it has been extensively studied to uncover interesting connections to organismal aging. Subjective age has been shown to be correlated with individuals’ health, mental state, cognitive function, longevity, socioeconomic status, and general well-being [6]. For example, higher subjective age is significantly associated with a decline in health, healthy behavior, and overall survival rate [7]. In addition, people with an older subjective age have been shown to have higher levels of systemic inflammation, as well as a risk of obesity, pulmonary and muscular dysfunction, and incidences of certain diseases [8–11].

The mechanisms that link age perception to these strictly biological phenomena are still only hypothesized. The socioemotional selectivity theory (SST) developed by Laura L. Carstensen at Stanford University, maintains that “the perception of time plays a fundamental role in the selection and pursuit of social goals” [12–14]. An extended perception of the personal timeline (lower subjective age) enables the long-term outlook and leads to more rational motivations and choices. Conversely, when the perception of one’s timeline is solely short term (higher subjective age), a person may choose more emotion-based options. This theory and the associated studies have highlighted the importance of the psychology of aging as a field of research and laid the foundation for studies of psychological and psychophysiological aging markers.

Subjective age is itself determined by various parameters, including personal experiences, social relationships, and cultural values [15, 16]. While some of them, such as genetics, are non-modifiable, many factors can be modified to influence subjective age. For example, physical activity, biomedical knowledge, psychological support pose as promising intervention targets.

Since personal attitude towards aging is strongly associated with the incidence of age-related diseases and mortality, modifying it through these gateways could be used to increase human healthspan. Consequently, large scale interventions might improve the global economy by promoting productive longevity in the developed countries experiencing population aging.

Historically, subjective age has been measured by asking study participants what age they felt were, what age group they identified with, or whether they felt older or younger than their chronological age [17]. Less frequently, a measure known as visually perceived age is used — the age estimated by independent observers using subject photographs. Visually perceived age has been used as a predictor of mortality in the Longitudinal Study of Aging Danish Twins [18]. Such direct ways of subjective and psychological age estimation are unfit to determine the intervention targets.

Recently, advances in artificial intelligence have permitted the identification of robust aging biomarkers to be used in the development of medical and lifestyle interventions. These biomarkers included those based on blood biochemistry [19, 20], transcriptomics and proteomics [21], epigenetic markers [22], microbiome [23], and photographs [24]. Such studies have facilitated the discovery and evaluation of new potentially geroprotective compounds along with therapeutic intervention strategies [25]. Researchers are now developing tools to accurately interpret biomarkers of aging known as “deep aging clocks” for applications in personalized medicine. Here, we propose that a similar technique can be used to identify biomarkers of psychological aging, which, in conjunction with the results obtained from biological aging studies, will have implications for industries, including the healthcare and consumer industries, among others [6, 26].

Deep aging clocks are broadly utilized in many applications in biomedical science, clinical research, the life insurance industry, and even consumer applications [5]. Here we have used a deep learning approach to identify predictors of both chronological age and subjective age. Using a Deep Neural Network (DNN)-based system, we have classified various features of human behaviors obtained from bio- and psycho-social questionnaires to predict the chronological and subjective age. Using the longitudinal dataset - Midlife in the United States (MIDUS, http://www.midus.wisc.edu/), which contains an extensive set of measures, including psycho-social, health, cognitive, biomarker, and neuroscience data (MRI, EEG), our DNN was capable of accurately predicting age. In addition to the robustness of this dataset, we chose MIDUS because of its high rate of inclusion in publications and funded studies.

Unlike biological data, such as gene expression or level of DNA methylation, the longitudinal survey data is easily interpretable using human intelligence and many of the survey questions are comprehensible even to non-professionals. However, human comprehension is different from the way machine learning algorithms (including DNN) interpret data vectors. To ensure that the final models can be further used in research and, possibly, in clinical practice, feature selection is necessary. Features that need to be used as psychosocial aging clock inputs should satisfy the following criteria:

1. *Modifiable*. Features that are predictive but not modifiable, such as the age of death of parents and/or grandparents, should be excluded. This increases the number of actionable items available to the model users;
2. *Non-leaking*. Age of children, years in retirement, and other demographic questions directly related to the chronological age were excluded. Such variables are trivial in interpretation and rarely lead to therapy targets. Moreover, they may obscure valuable, therapeutic trends by having disproportionately large importance scores;
3. *Predictive*. A feature needs to be associated with age-related changes. Selecting predictive features can be realized as an iterative process, in which feature importance analysis is carried out after each round of model training. In the case of MIDUS 1 most predictive variables were associated with health, personality traits, occupation and other psychosocial aspects of life.
4. *Robust*. Aging clock features need to be predictive across different demographics, as well as in the same person at different time points;
5. *Non-collinear*. Features that are strongly correlated with each other should be reduced. For example, such variables as weight, body mass index, and waist circumference are strongly correlated and add little predictive value when present in an aging clock all at once.

**Figure 1:**
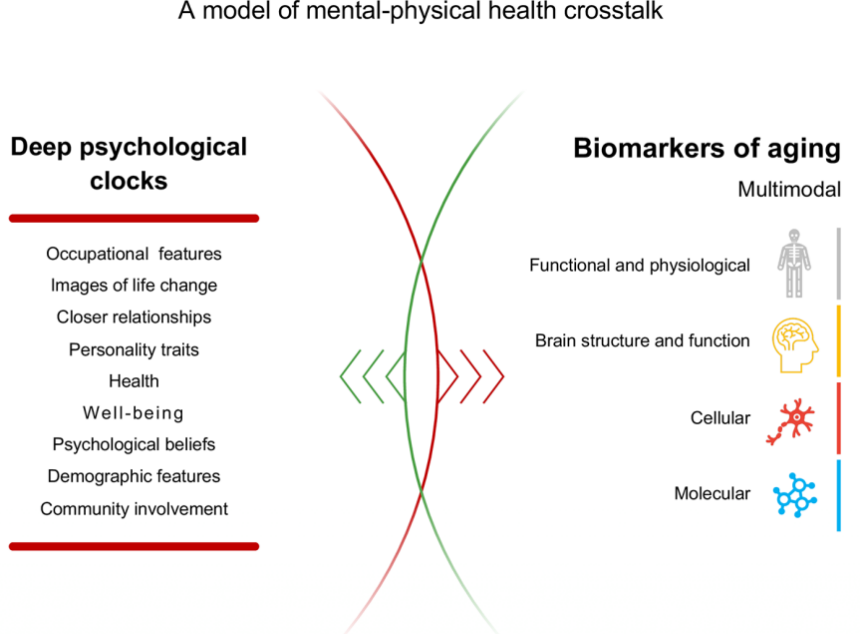
**A model of mental-physical health crosstalk.** One’s mindset may determine the decisions that ultimately affect their health. Interpreting the biological consequences of lifestyle choices in terms of aging has become relatively easy thanks to a wide variety of aging clocks. The psychological drivers behind these choices, however, are poorly understood. For example, it remains unknown how agreeableness or feeling fragile affects mitochondrial upkeep and DNA methylation profiles. The feedback loops that go from molecular level aging biomarkers back to psychological traits are yet another gap in our understanding of aging mechanisms. Deep psychological clocks may help us bridge this gap and bring psychology into the domain of biogerontological studies.

Based on these guidelines, we selected 50 features as psychological aging biomarkers. Our PsychoAge model was trained to predict chronological age based on these parameters in a cohort of mentally healthy people, while SubjAge was trained to approximate the subjects’ perceived age. In the end, the mean absolute error (MAE) of our models achieved 6.7 years for PsychoAge and 7.3 years for SubjAge. The subsequent feature important analysis allowed us to rank the features and select the most potent markers of aging among them. We believe that this project can serve as a foundation stone for a mental-physical health crosstalk model (Figure 1).

## RESULTS

### Psychological and subjective age estimation

To develop psychological clocks, we trained two DNN’s using anonymized surveys from MIDUS 1 — PsychoAge and SubjAge. The former was designed to estimate human chronological age based on a set of 50 psychosocial features, and the latter — human subjective age using the same set of features.

**Figure 2:**
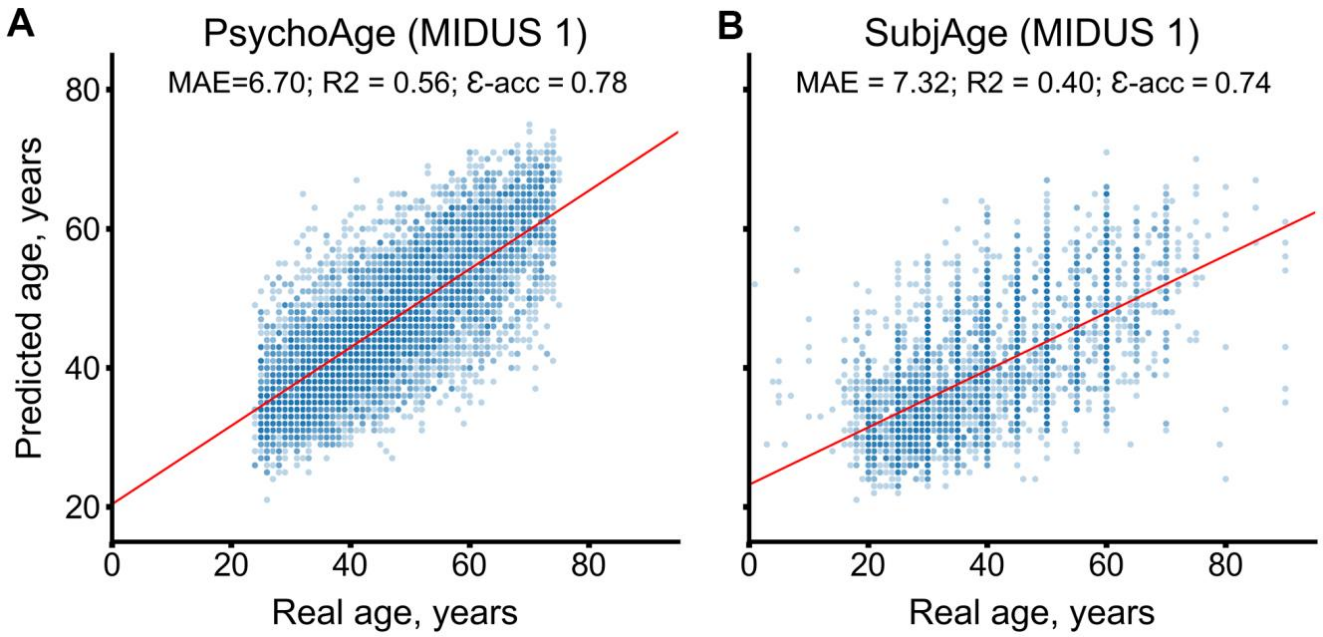
**PsychoAge and SubjAge provide better than baseline estimates of chronological age and subjective age, respectively, in MIDUS 1 (N samples = 6071).** (**A**) PsychoAge chronological age predictions in MIDUS 1 (MAE = 6.70 years; epsilon accuracy = 0.78). (**B**) SubjAge subjective age predictions in MIDUS 1 (MAE = 7.32 years; epsilon accuracy = 0.74).

During CV, PsychoAge reached an MAE of 6.70 years and an epsilon accuracy of 0.78, while SubjAge reached an MAE of 7.32 years and an epsilon accuracy of 0.74 (Figure 2). Both PsychoAge and SubjAge outperformed baseline median age assignment in MIDUS 1 (N_samples_=6071). When verified in MIDUS 2 and MIDUS Refresher, these quality scores dropped, although remained better than baseline values (Table 1).

**Table 1:** PsychoAge and SubjAge outperform baseline median age assignment in all datasets used for this work.

	**Dataset**	**Psychological age**		**Subjective Age**		**N, people**
		**MAE**	**ε-accuracy**	**MAE**	**ε-accuracy**	
**CV**	MIDUS 1	6.70	0.78	7.32	0.74	6071
	MIDUS 1 (Baseline)	10.79	0.49	9.78	0.56	
**Verification sets**	MIDUS 2	7.18	0.73	8.53	0.66	3870
	MIDUS 2 (Baseline)	10.27	0.52	10.72	0.52	
	MIDUS Refresher	7.73	0.70	8.56	0.65	2521
	MIDUS Refresher (Baseline)	12.36	0.39	11.27	0.34	

Metrics computed for MIDUS 1 are derived from per fold CV predictions. MAE stands for “Mean Absolute Value”; CV stands for “cross-validation”.

We further tested the efficiency of our approach on significantly smaller samples by dividing MIDUS 1 into age groups based on chronological age (25-39, 40-64, 65-75 years) and training new DNNs for each one of them with the same set of 50 variables. Among the chronological age predictors, only the one trained on 682 samples from the 65-75 years group showed worse than baseline performance. Meanwhile, among the subjective age predictors only the 25-39 years DNN performed worse than baseline.

Lower chronological age estimation accuracy in the elderly may be attributed to greater lifestyle homogeneity in this cohort. We also hypothesize that younger people have a more fluid concept of aging compared to older adults, which might have affected the 25-39 cohort model performance. These hypotheses, however, were not tested in this work and need to be addressed in a follow-up project.

**Figure 3:**
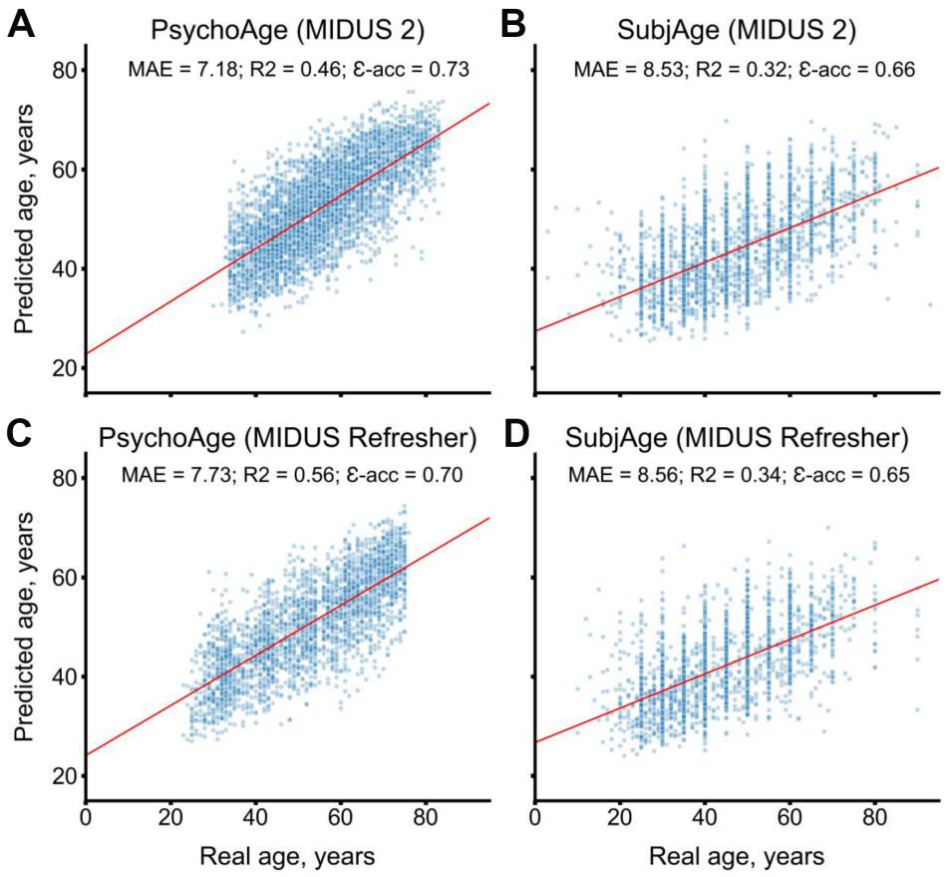
**PsychoAge and SubjAge provide better than baseline estimates for chronological age and subjective age, respectively, in MIDUS 2 (N samples = 3870) and in MIDUS Refresher (N = 2521).** (**A**) PsychoAge chronological age predictions in MIDUS 2 (MAE = 7.18 years; epsilon-accuracy = 0.73). (**B**) SubjAge subjective age predictions in MIDUS 2 (MAE = 8.53 years; epsilon accuracy = 0.66). (**C**) PsychoAge chronological age predictions in MIDUS Refresher (MAE = 7.73 years; epsilon-accuracy = 0.70). (**D**) SubjAge subjective age predictions in MIDUS Refresher (MAE = 8.56 years; epsilon accuracy = 0.65). Red lines mark ordinary least squares regressions. R2 stands for “coefficient of determination”, MAE stands for “Mean Absolute Error”, ε-acc stands for “epsilon-accuracy”.

MIDUS 2 (N=3870 respondents) and MIDUS Refresher (N=2521 respondents) datasets were used for model validation. Both models remained accurate when tested in these datasets (Figure 3). PsychoAge showed an MAE of 7.18 and 7.73 years, respectively, while SubjAge — an MAE of 8.53 and 8.56 years, respectively.

The predictions displayed in the scatter plot were obtained during CV. Red lines mark ordinary least squares regressions. R2 stands for “coefficient of determination”, MAE stands for “Mean Absolute Error”, ε-acc stands for “epsilon-accuracy”.

### Psycho-social feature importance analysis

**Figure 4:**
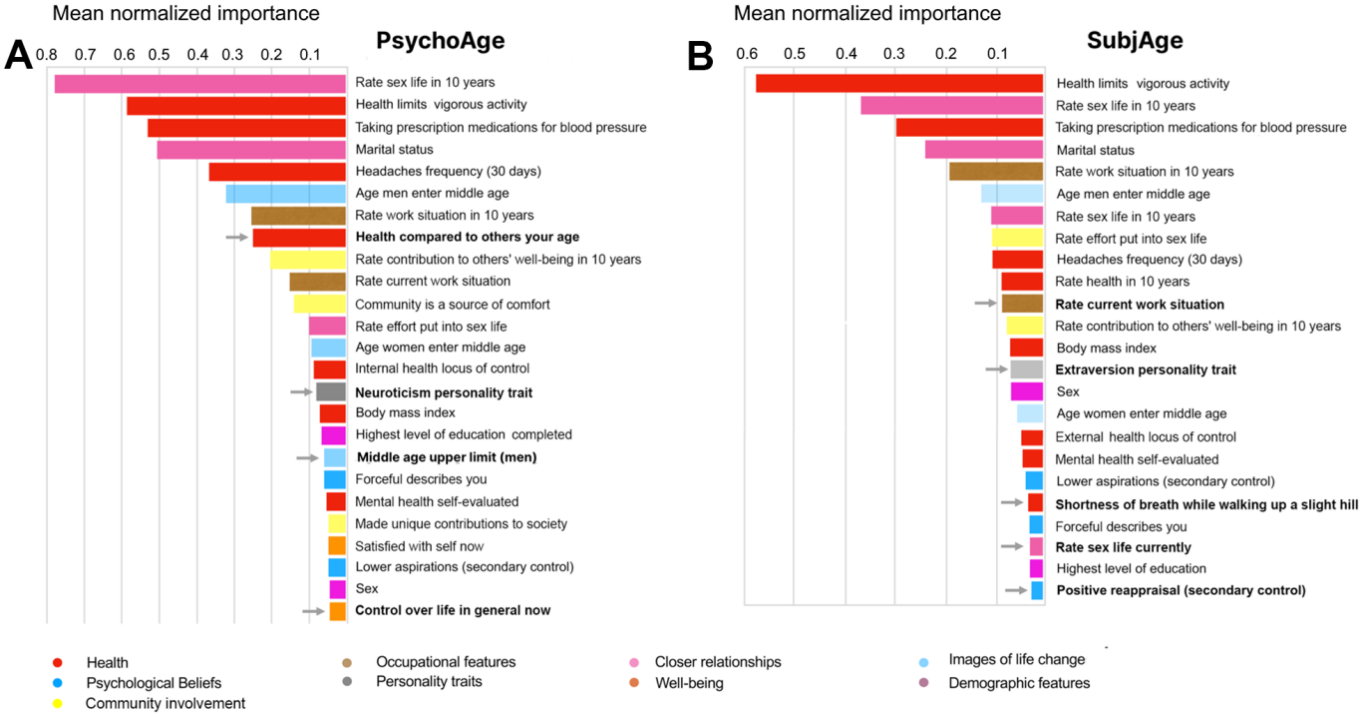
**PsychoAge and SubjAge use the same variables to predict chronological and subjective age, but assign different importance to them.** (**A**) Top-25 important features for estimating chronological age with PsychoAge. Features not present in the SubjAge top-25 list (marked by arrows): “health compared to others your age”, “neuroticism personality trait”, “middle age upper limit (men)”, “control over life in general now for psychological age prediction. (**B**) Top-25 important features for estimating subjective age with SubjAge. Features not present in the PsychoAge top-25 list (marked by arrows): “rate current work situation”, “extraversion personality trait”, “openness personality trait”, “shortness of breath while walking up a slight hill”, “rate sex life currently”, “positive reappraisal (secondary control)”. Mean importance is the normalized mean of PFI and DFS importance scores.

We explored the importance of the features used by PsychoAge and SubjAge using PFI and DFS techniques on MIDUS 1. Scores produced by them were normalized and averaged to yield two feature lists in which all features were ranked according to the magnitude of their effect on model output (Figure 4).

The most important PsychoAge and SubjAge features belonged to the categories of closer relationships and health. More specifically, top-5 important features in both these aging clocks contained variables describing “rate sex life in 10 years” and “marital status” (closer relationships category), as well as “health limits on vigorous activity”, “prescription medications for blood pressure” (health category). The “headaches frequency (30 days)” variable was ranked fifth most important for PsychoAge, while in SubjAge it was ranked only ninth.

Interestingly, the top-25 feature lists are quite dissimilar between PsychoAge and SubjAge. We explored these dissimilarities to see which features determine the difference between psychological aging and human idea of it (subjective aging). Variables such as (i) “health compared to others your age”, (ii) “neuroticism personality trait”, (iii) “middle age upper limit (men)”, and (iv) “control over life in general” are identified as important only in PsychoAge (Figure 4A). Conversely, some variables were important only for SubjAge predictions: “rate current work situation”, (ii) “extraversion personality trait”, (iii) “openness personality trait”, (iv) “shortness of breath while walking up a slight hill”, (v) “rate sex life currently”, and (vi) “positive reappraisal (secondary control)” (Figure 4B).

Note that neuroticism was the only big five personality trait present in the top-25 features for PsychoAge. In the meantime, openness and extraversion are the only big five personality traits important for SubjAge prediction. This finding can be interpreted as neurotic tendencies being inherent to psychological aging, while changes in openness and extraversion are much less significant parts of this process. They, however, greatly affect the personal perception of age.

Feature importance analysis with PFI and DFS is sufficient to determine the significance of features in absolute terms, but it does not convey any information on the direction of change. Other methods should be employed to associate psychological aging, for example, with increasing or decreasing neuroticism (see “Results: Psychological aging across different age groups”).

### Psychological aging across different age groups

### Psychological aging core and uniquely important features

To describe how different variables contribute to psychological aging throughout human life, we trained DNNs on MIDUS 1 age group subsamples (25-39, 40-64, 65-75 years). These models contained the same 50 features as PsychoAge and Subjage, but their relative importance was not constant. In other words, a variable’s contribution to psychological aging was not static and its influence may vary with time.

First, we explored important variables (top-25 mean normalized PFI and DFS scores) shared by all age-group specific clocks to define the “psychological aging core” — features that significantly shift throughout one’s lifespan (Table 2). Core features that determined the chronological aspect of psychological aging in MIDUS 1 contained neuroticism, seeing the community as a source of comfort and defining the lower boundary of male middle age. These features are expected to have life-long trends that let DNNs tell an old and a young person apart. Personality traits that were rendered important for human perception of age included aspirations scale, extraversion, openness, being career-oriented, and the prevalence of the positive reappraisal coping mechanism. Changes in these traits are expected to drive the internal psychological clock in humans of all ages.

**Table 2:** Features important for subjective and chronological age prediction differ across age groups in MIDUS 1.

			**Training set**		
**Age group**			**[25-39]**	**[40;64]**	**[65;75]**
**N, people**			**2040**	**3339**	**682**
**Target variable**	**Chronological age**	**Unique important features**	Effort put in life overall; Lower aspirations; Forcefulness; Current opinion about contributing to the well-being of others.	Define age women enter middle age; Middle age upper limit (male); Current opinion about work situation; Contributing to the well-being of others in 10 years; Made unique contributions to society; Current opinion about life overall; Current over life in general at present; Current opinion about sexual aspects of life.	Live for today; Persists in goal striving; Positive re-appraisal; Highest level of education; Agency; Current opinion about health.
		**Core features**	Now taking prescription medications for blood pressure; Marital status;	3. Headaches frequency (30 days); 4. Body mass index; 5. Define age when men enter middle age;	6. Community is a source of comfort; 7. Neuroticism;
		**MAE, years**	3.35	4.83	2.45
		**Baseline MAE, years**	3.56	6.00	2.39
		**ε-accuracy**	1.00	0.91	1.00
	**Subjective age**	**Unique important features**	Conscientiousness; Opinion about control of life in general; Define age women enter middle age; Mental health (self-evaluated);	Sex life expectations in 10 years; Health locus of control – others; Highest level of education; Contributing to the well-being of others in 10 years; Chronic conditions (12 months).	Optimism; Effort put in contributing to the well-being of others in 10 years; Opinion about life in general; Effort put in health; Outgoing; Sex life; Neuroticism.
		**Core features**	Body mass index; Lower aspirations;	3. Extraversion; 4. Rate work situation in 10 years;	5. Effort put in work; 6. Openness; 7. Positive reappraisal.
		**MAE, years**	5.23	6.89	7.63
		**Baseline MAE, years**	5.04	7.40	8.07
		**ε-accuracy**	0.88	0.76	0.72

A series of DNNs was trained using samples from specific age groups (25-39, 40-64, 65-75 years) to inspect which features get recognized as important only within these groups. Features used by any model are the same 50 features as in SubjAge and PsychoAge; unique important features are defined as the features present in the top-25 importance list for only one aging clock. MAE stands for “Mean Absolute Error” See Supplementary Table 2 for a more detailed report.

The psychological core defines what aspects of human personality are constantly evolving and thus qualify as lifelong markers of psychological aging. But some features may quickly shift in a certain life period and be important for measuring psychological aging in this period only. These variables are called “uniquely important” in Table 2. They identify which aspects of psycho-social life change the most reliably within an age group and thus get assigned higher importance within the corresponding age predictor.

To illustrate, agency is a uniquely important feature for accurate chronological age estimation in elderly people. This does not mean that being more or less willing to shape one’s own life is a trait only seen (or uniquely lacking) in the elderly. Most probably, agency was considered important in this age group, since people experience a major shift in this psychological attribute while going from 65 to 75 years. The DNN may have learned this possibly non-linear pattern to move a person closer to the upper or the lower boundary. In contrast, younger people may maintain more consistent agency throughout their lives, which makes DNNs seek aging-related patterns in other features.

Other variables recognized as uniquely important in Table 2 should be interpreted in a similar fashion. They represent the variables that go through a major shift within the specified age range, yet the direction of this shift cannot be determined using PFI or DFS techniques only. We address the directionality of age-related changes in the next section.

Apart from agency, other psychological traits such as positive reappraisal, persistence in goal striving, and living for today were found to be key features in the older generation. Meanwhile, aspirations scale and forcefulness turned out to be more significant attributes for accurate chronological age prediction in the 25-39 years cohort.

Among the big five personality traits, neuroticism was found to be important for subjective age prediction in the 65-75 years cohort, while in the 25-39 years cohort conscientiousness was a unique significant big five trait. Interestingly, optimism is also considered important to accurately estimate the subjective age in the 65-75 years cohort.

Contrary to the other age groups, psychological aging in the middle-aged (40-64 years) people were driven not by personality traits, but by the measures of social success and expectations of future life: overall education, career satisfaction now and in 10 years, having any unique contributions to the public good, as well as the expected contribution towards the well-being of others in 10 years.

Some health-related features also differed in significance across age groups. For example, self-evaluated mental health was uniquely important for accurate subjective age prediction in the 25-39 years cohort. Current opinion on health was uniquely important for accurate chronological age prediction in the 65-75 years cohort.

### Directionality of age-related changes

To interpret the available variables in terms of the effect they have on psychological aging, we employed an approach based on linear models with mixed effects (Table 3). Most variables used by PsychoAge and SubjAge have a discordant effect on the predictions in MIDUS 1. In other words, variables associated with higher PsychoAge are also associated with lower SubjAge.

**Table 3:** Most variables used by PsychoAge and SubjAge have a discordant effect on predictions.

**Fixed variables**	**Range**	**Effect on SubjAge**		**Effect on PsychoAge**	
		**Coef**	**std**	**Coef**	**std**
**Health**					
Shortness of breath while walking up a slight hill	1-Yes,2-No	-2.91*	0.12	0.88*	0.16
Taking prescription medications for blood pressure	1-Yes,2-No	-2.62*	0.18	-3.12*	0.22
Health limits vigorous activity	1-a lot,4-not at all	-2.05*	0.05	0.19	0.08
Mental health self-evaluated	1-poor,5-perfect	-1.41*	0.06	0.95*	0.08
Rate current health	0-worst,10-best	-1.16*	0.04	0.93*	0.06
Rate health in 10 years	0-worst,10-best	-1.02*	0.04	0.50*	0.05
Internal health locus of control	1-low, 7-high	-0.94*	0.07	0.71*	0.09
Headaches frequency	1-every day,6-never	-0.62*	0.04	1.29*	0.05
Effort put in health	0-none,10-very much	-0.32*	0.03	0.61*	0.04
Body mass index	kg/m2	0.17*	0.01	-0.07*	0.01
Health compared to others your age	1-better,5-worse	1.91*	0.06	-2.14*	0.08
Having any chronic conditions, 12 months	0-No, 1-Yes	2.14*	0.13	-0.20	0.17
External health locus of control	1-low, 7-high	0.38*	0.04	0.18*	0.05
**Personality traits**					
Openness personality trait	1-low, 4-high	-2.41*	0.10	1.76*	0.14
Extraversion personality trait	1-low, 4-high	-2.35*	0.10	2.24*	0.13
Agreeableness personality trait	1-low, 4-high	-1.28*	0.12	1.90*	0.15
Agency personality trait	1-low, 4-high	-1.18*	0.09	1.08*	0.11
Conscientiousness personality trait	1-low, 4-high	-1.86*	0.13	2.03*	0.16
Neuroticism personality trait	1-low, 4-high	1.72*	0.08	-2.40*	0.10
**Psychological Beliefs**					
Persist in goal striving (primary control)	1-a lot, 4-not at all	-1.89*	0.10	2.37*	0.13
Positive reappraisal (secondary control)	1-rarely, 4-often	-1.77*	0.09	2.08*	0.12
Lower aspirations (secondary control)	1-rarely, 4-often	1.27*	0.10	-0.26	0.13
Optimistic describes you	1-a lot, 4-not at all	1.29*	0.07	-1.39*	0.09
Forceful describes you	1-a lot, 4-not at all	0.35*	0.06	-0.55*	0.08
Live for today	1-disagree 4-agree	0.68*	0.08	0.04	0.11
Outgoing describes you	1-a lot, 4-not at all	0.88*	0.07	-0.97*	0.09
**Well-being**					
Rate life overall	0-worst, 10-best	-0.57*	0.04	0.86*	0.05
Effort put in life overall	0-worst, 10-best	-0.38*	0.03	0.33*	0.04
Control over life in general now	1-a lot, 4-not at all	1.30*	0.10	-1.91*	0.12
Satisfied with life at present	1-a lot, 4-not at all	1.44*	0.08	-2.01*	0.10
Satisfied with self at present	1-a lot, 4-not at all	1.61*	0.09	-2.08*	0.11
**Occupational features**					
Rate work situation in 10 years	0-worst, 10-best	-0.57*	0.02	0.03	0.03
Effort put in work	0-worst, 10-best	-0.35*	0.02	0.08	0.03
Rate current work situation	0-worst, 10-best	-0.42*	0.03	0.69*	0.03
**Closer relationships**					
Rate sex life in 10 years	0-none, 10-very much	-0.48*	0.02	-0.37*	0.03
Rate effort put into sex life	0-none, 10-very much	-0.39*	0.02	-0.11*	0.03
Rate sex life currently	0-worst, 10-best	-0.32*	0.02	-0.07	0.03
Marital status	1–married, 2-5–not married	-0.18*	0.04	-0.22*	0.05
**Community involvement**					
Rate contribution to others’ welfare in 10 years	0-worst, 10-best	-0.19*	0.03	-0.09	0.04
Community is a source of comfort	1-agree,7-disagree	0.13*	0.04	-0.81*	0.05
Rate current contribution to others’ well-being	0-worst, 10-best	-0.13*	0.03	0.21*	0.04
Effort put into others’ well-being	0-none, 10-very much	-0.12*	0.03	0.26*	0.04
Made unique contributions to society	1-a lot, 4-not at all	0.23*	0.06	-0.88*	0.08
World is becoming a better place	1-agree, 7-disagree	0.19*	0.04	-0.44*	0.05
**Demographic features**					
Highest level of education completed	1-no school, 12-PhD	-0.17*	0.03	0.10*	0.03
**Images about life change**					
Middle age upper limit (women)	years	-0.03	0.01	0.18*	0.01
Middle age upper limit (men)	years	-0.03	0.01	0.19*	0.01
Age women enter middle age	years	-0.02	0.01	0.25*	0.01
Age men enter middle age	years	-0.01	0.01	0.31*	0.01

Variables that increase SubjAge typically also decrease PsychoAge. Directional variable effects were estimated using linear models with random intercepts (see Figure 6 and Supplementary Figure 2).

Thirteen health-related variables were tested, among them all were significantly (effect coefficient above 3σ) associated with SubjAge, but only 11 — with PsychoAge. Having chronic conditions, as well as being physically challenged, did not affect PsychoAge prediction. But these specific health problems were more frequent in those who felt older than their actual age.

Variables corresponding to other health problems, such as headaches, hypertension, shortness of breath, and obesity displayed a pattern in which less severe conditions decreased SubjAge and increased PsychoAge. Similar patterns were observed for the self-evaluated mental and physical health scores.

**Figure 5:**
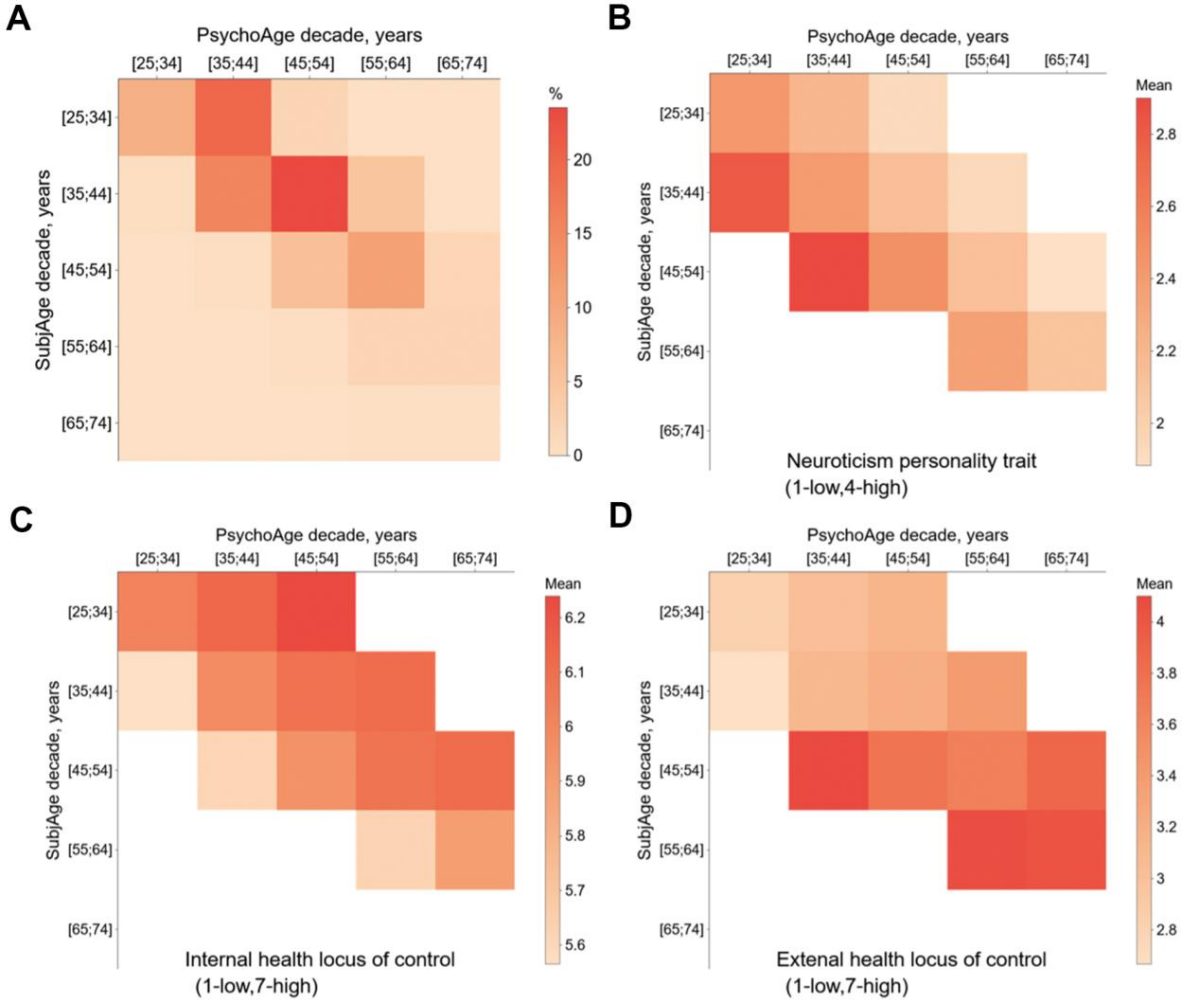
**Mixed-effects linear models were used to observe how changing a variable affects PsychoAge and SubjAge predictions in MIDUS 1.** (**A**) Confusion matrix of MIDUS 1 samples divided based on their PsychoAge and SubjAge predictions (see Supplementary Figure 1 for SubjAge × Real Age and PsychoAge × Real Age confusion matrices). (**B**–**D**) Heatmaps of mean values for three important features (neuroticism, internal and external locus of health control) in SubjAge × PsychoAge confusion groups. Groups with <25 samples were excluded from the mixed-effects analysis and left blank on the Confusion matrices for all psychosocial variables are listed in Supplementary Figure 2.

Among the health-related features, external locus of health control was the only feature that was significantly increased in people with both high SubjAge and high PsychoAge (Figure 5). Noteworthy, its linearly positive effect on SubjAge was twice as large. Internal locus, being a by definition opposite health mindset, was not associated with lower PsychoAge. It was, however, associated with lower SubjAge. Internal locus thus showed behavior typical of most other health-related variables, in which less severe problems were associated with lower SubjAge and higher PsychoAge.

Most tested personality traits and beliefs also displayed a connection to psychological aging. All traits from the big five, except for neuroticism, (openness, conscientiousness, extraversion, agreeableness) were associated with higher PsychoAge and lower SubjAge. Neuroticism had an opposite effect on age prediction: decreasing PsychoAge and increasing SubjAge (Figure 5).

Other variables that describe whether a person is optimistic and proactive showed a pattern similar to the four out of five big five traits. The prevalence of positive reappraisal, being outgoing, optimistic, forceful, and goal-oriented decreased SubjAge and increased PsychoAge in the mixed-effects models. A similar pattern was observed for the optimistic beliefs that “community is a source of comfort” and that “the world is becoming a better place”. Meanwhile, lower aspirations and “live for today” attitude only increased SubjAge with no significant effect on PsychoAge.

Another set of beliefs we tested concerned the question of when middle age starts and ends. People who were predicted to be older by PsychoAge responded to specified higher boundaries of this life period. For example, people whose PsychoAge matched their SubjAge estimated male middle age period to start at 39-47 years on average. In the meantime, people from the same SubjAge groups but with PsychoAge 20 years larger suggested that men’s middle age started at 45-50 years. Despite the large effect on PsychoAge, the concept of middle age did not affect SubjAge. People from the same PsychoAge group were extremely consistent in their definitions of male and female middle age, even when coming from different SubjAge cohorts.

The last 17 features not yet discussed belonged to the categories of personal well-being, accomplishment, and close relationships. Variables describing marital status and sex life were a rare occurrence of concordant effect on both PsychoAge and SubjAge. More fulfilling sex life or being married decreased the predictions of both these aging clocks.

The level of life satisfaction and personal accomplishment correlated with lower SubjAge, and in some cases — with higher PsychoAge. Among the variables that did not affect PsychoAge were effort put into work, expectations of work, and contributing to the well-being of other people in 10 years.

### Psychological aging and mortality

**Figure 6:**
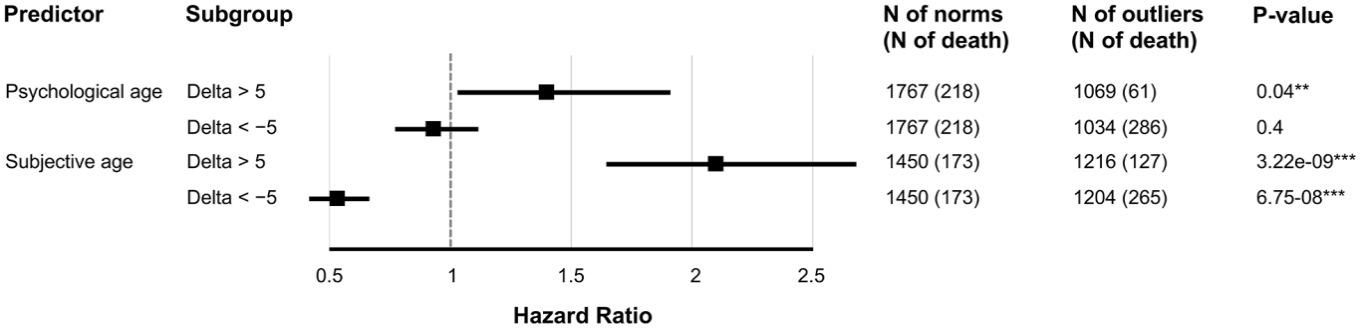
**SubjAge is a more significant all-cause mortality risk factor than PsychoAge.** Hazard ratios were obtained with Cox regression. Delta is the difference between actual age (chronological for PsychoAge or subjective for Subjective) and their DNN-derived estimations. Each row represents a hazard ratio and the 95% confidence interval associated with a specific feature. Note: “***” for P-value of 0.001; “**” for P-value of 0.01; “*” for *P*-value of 0.05.

We inspected whether SubjAge and PsychoAge prediction errors were indicative of all-cause mortality risk using Cox regression. In models adjusted for gender, age, and either PsychoAge or SubjAge error both predictors were shown to be significant (*p-value*<0.05) risk factors (Figure 6). SubjAge delta above +5 years was associated with a more than two-fold increase in mortality rate (HR=2.11), and SubjAge delta below -5 years was recognized as a major protective factor (HR=0.54). HRs produced by the PsychoAge survival model were much less significant and carried less weight: HR for PsychoAge delta above +5 years was 1.14 (*p-value*=0.04) and HR for delta below -5 was 0.93 (*p-value*=0.40).

**Figure 7:**
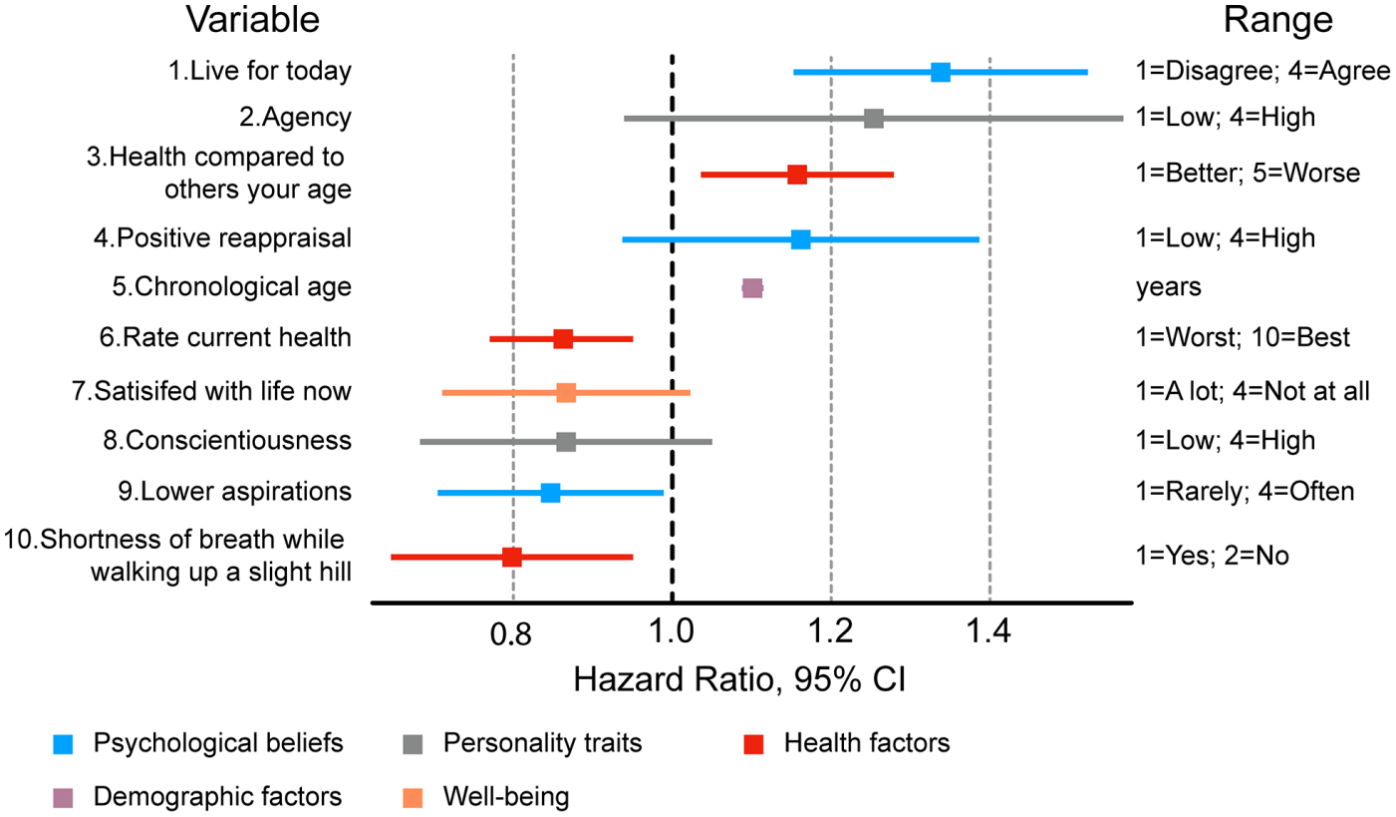
**Ten most important features associated with mortality risk in a Cox regression adjusted for 49 psychosocial variables, sex, and chronological age.** Most significant risk factors include variables describing personality traits and health status.

We then compared the 50 features used by both PsychoAge and Subjage plus chronological age within one Cox regression (Figure 7 and Supplementary Figure 2). The 10 most powerful risk factors based on HR magnitude belonged to the categories of:

- Health (“Rate current health”, “Health compared to others your age”, “Shortness of breath while walking up a slight hill”);
- Personality (“Conscientiousness”, “Agency”);
- Psychological beliefs (“Live for today”, “Positive reappraisal”, “Lower aspirations”);
- Personal well-being (“Satisfied with life at present”);
- Demographics (“Chronological age”).

Among these risk factors only “Live for today”, “Health compared to others your age”, “Chronological age”, “Rate current health”, “Lower aspiration” and “Shortness of breath” were deemed significant.

Surprisingly, the optimistic outlook associated with higher “Live for today values” was the largest contributor to the mortality risk (HR=1.35), while lower aspirations were a significant mortality protector variable (HR=0.84). These results, however, are in line with the previous findings in the mixed-effects analysis, in which both these features were shown to increase SubjAge, while only insignificantly affecting PsychoAge.

“Health compared to others your age” also increased SubjAge (coef = 1.91) but also decreased PsychoAge predictions (coef = -2.14). Despite this compensatory effect of reduced PsychoAge, “Health compared to others your age” (1=”Much better”, 6=”Much worse”) was recognized as a significant risk factor (HR=1.16). This may be attributed to the previously established higher mortality associated with increased SubjAge predictions (Figure 6). “Rate current health” behaved similarly, although due to its reversed scale (0=”Worst”, 10=”Best”) its HR was below one — 0.86.

Significantly low HR for “Shortness of breath” may also be misleading, since it is a binary variable, in which “1” signifies no problems with breathing, while “2” — shortness of breath when walking uphill. In the mixed-effects analysis having short breath was associated with, on average, 2.91 years larger SubjAge predictions and only 0.88 years smaller PsychoAge predictions. This furthers the point that the most significant all-cause mortality risk factors are associated with disproportionately larger SubjAge predictions.

Column “range” describes the range of possible values for each variable. For a full list of hazard ratios and associated MIDUS variable names see Supplementary Figure 3.

## DISCUSSION

In this article, we present two novel aging clocks created within the deep learning paradigm — PsychoAge and SubjAge. Both these clocks use the same set of 50 psychosocial features to estimate human chronological age and subjective age, respectively. These clocks showed superior performance during CV in MIDUS 1 (MAE_PsychoAge_= 6.70 years; MAE_SubjAge_= 7.32 years) and were verified in two other large data sets — MIDUS 2 and MIDUS Refresher (Table 1). In terms of epsilon accuracy, PsychoAge reached a score of 0.78 in MIDUS 1, and SubjAge — 0.74.

Having trained and verified the final models, we aimed to understand how PsychoAge and SubjAge see human aging and what features they pay the most attention to. With a tandem PFI-DFS approach we ranked all features according to their relative importance. Top-5 important features in both clocks were associated with health conditions (e.g. headache frequency) and relationship status (marital status, expectations from sex life in 10 years). Less significant features greatly differ in their relative importance for SubjAge and PsychoAge predictions. For example, top-20 PsychoAge features contain only one personality trait — neuroticism. Meanwhile, the only personality traits encountered among top-20 SubjAge features are — extraversion and openness.

These three personality traits, along with conscientiousness and agreeableness form “the big five traits”, which are commonly used in practice and scientific research to describe the human mental state landscape. High neuroticism is characteristic of emotional instability and common mental disorders, such as mood disorders, anxiety, and substance use disorders. Openness and extraversion, on the other hand, are considered more balanced traits, although their abnormally low scores are also related to social phobia and agoraphobia [27]. Positive orientation, seeking warmth, social interaction, and emotional stability may play an important role in psychological aging.

We hypothesized that the human mind evolves throughout the lifespan, which results in some traits, beliefs, or priorities shifting — not always in unison or at the same speed. At certain life stages, career-related priorities may rise, while at others they may fade and be replaced by different priorities. These lifelong progressions of the psyche eventually get recognized by the neural networks we constructed to let them build an image of psychological aging.

This idea of human mind progression is described in much more detail in the review of SST by Laura Carstensen. SST suggests that younger people are more goal-oriented, interested to obtain new knowledge and skills, while older people tend to value emotionally meaningful goals more.

To identify the psychosocial features that change while a person advances from one age group to another we trained separate DNNs on MIDUS 1 samples from three age groups (25-39, 40-64, 65-75 years). First, we defined the psychological aging core — variables that remain highly important (top-25) across all age groups (Table 2). The core contained not only strictly psychological features, however. To illustrate, marital status, hypertension medication, headaches, and body mass index were among the seven core features required for accurate chronological age prediction. Interestingly, neuroticism score also belonged to the same psychological aging core, as well as seeing the community as a source of comfort. Psychological traits within the subjective aging core contained aspirations scale, extraversion, openness, positive reappraisal prevalence, and two career-related variables — effort put into work now and work expectations in 10 years. In contrast to the first psychological core, which contained few psychological traits, the subjective core consisted almost exclusively of psychological features.

This highlights an important distinction between aging *per se* (as judged by PsychoAge) and our perception of it (as judged by SubjAge): subjective aging is mostly dependent on internal causes.

We also explored the uniquely important features for each age group — features that emerged only in one top-25 set. Since these features were recognized as important only in these groups, it may be assumed that they shift the most markedly during the corresponding life periods. To illustrate, young adults were not the only age group who responded affirmatively to the statement “Forceful describes you well”, but rather many of these people went through a transformation that affected their forcefulness. Detecting such a change was essential for a predictor to accurately predict whether a person was at the beginning or the end of this phase of life.

On their own, DNNs are unable to tell generational and age-related changes apart or tell the difference between pro-longevity and progeroid features. Thus, the results of the feature importance analysis should always be cautiously inspected and verified in more rigorous settings. Still, feature importance analysis is a powerful tool for hypothesis generation and the verification of overall biological relevance.

While neuroticism was identified as a part of the psychological aging core, it was also a uniquely important subjective aging feature in the elderly MIDUS 1 subsample. This may be interpreted as neuroticism progressing unnoticed by an individual until old age when it starts to affect the perception of age. Previous studies identified that neuroticism tends to cause low emotional differentiation, anxiety, and depression in old people [28, 29].

Other personality traits rendered important for subjective age estimation in the elderly were optimism, being outgoing, and content with life in general. These results indicate that these might be top-priority features to focus on while developing policies aimed to involve the elderly in social life. Several studies have shown the importance of a social and productive lifestyle during aging [30, 31]. psychologically important and active events may protect against aging diseases, such as dementia [32].

After establishing which variables are important in absolute terms, we aimed to measure the models’ response to changes in their values. Using mixed-effects linear models, we explored the monotonic trends between 50 variables, PsychoAge and SubjAge predictions (Supplementary Figure 1 and Supplementary Figure 2).

Once again, neuroticism showed unique behavior. Contrary to the other big five traits, neuroticism score was associated with higher SubjAge and lower PsychoAge. More specifically, people within the same PsychoAge group could have >5 years of SubjAge difference due to differences in neuroticism score alone. This verifies our previous conjecture that neuroticism is a key marker of subjective aging and may be used as a sensitive measure of emotional states and late-life depressive symptoms.

Other big five traits also had significantly large effects on both PsychoAge and SubjAge. For example, a person with the bottom openness score would feel 7.2 years older than their PsychoAge counterpart with the top score. In the meantime, a person with the bottom openness score would be 5.3 years younger, as measured by PsychoAge, than their SubjAge counterpart with the top score. Similar tendencies could be observed for most other personality traits, thus building a strong case for SST.

Interestingly, personal opinion on when middle age starts and ends was significantly associated with higher PsychoAge but does not affect SubjAge. We hypothesize that this is an indication of “time dilation” associated with aging. As people get older, they place “middle age” higher and higher, as if their lifetime dilates, while younger participants may have stereotypes about aging and place “middle age” lower. An excellent study on the topic of perception of age stereotypes and self-perception of aging has been written by Hummert [33].

Among the health-related features, the distinction between internal and external health locus of control is of utmost interest. Health locus of control is a set of personal beliefs and experiences that determine whether a person takes responsibility for their health (internal locus) or considers it to be outside of their power, fully dependent on external factors (external locus). Internal locus of control is associated with a problem-solving mindset, while external locus is tied to depression, anxiety, and suicidal thoughts, as well as maladaptive behaviors [34]. We demonstrated that external locus of health control is a rare feature that demonstrated a linearly positive effect on both PsychoAge and SubjAge. It was the only feature to offer no payoff in at least one aging dimension, except for “Taking prescription medications for blood pressure”. Internal control, *per contra*, did not display concordant linearly negative effect on. Instead, it decreased SubjAge and decreased PsychoAge, just as most other health-related variables.

While the external locus of control was a senopositive (higher values increase age predictions) feature in both aging dimensions, some features were identified as double senonegative (higher values decrease age predictions). Increasing values for the variables from the relationships category were associated with lower PsychoAge and SubjAge, thus favoring single people content with their sex life, who expected to remain sexually active in 10 years. In this case, it is difficult to conclude the cause-effect relation between psychological aging and sexuality. Is reduced libido a precondition to becoming subjectively old? Or does feeling old due to other factors make people less interested in the sexual aspect of life? Can more satisfying sex life prolong healthy longevity, or does PsychoAge simply see higher sex drive as a feature more frequently encounter in the youth? More thorough research is required to answer these questions as well as similar questions concerning other variables.

Although the effect of most variables on PsychoAge and SubjAge was shown to be discordant, the magnitude of their effects on these two measures of psychological aging is not equal. Since the target variable in the mixed-effects model is expressed in years, Table 3 can be used to approximate how a shift in a psycho-social parameter will affect PsychoAge or SubjAge, and which one of them will change more. For example, the variable “Rate health in 10 years” is a survey question that measures health expectation on a scale from 0 to 10, from worst to best. Each increment increases PsychoAge by 0.5 years but also decreases SubjAge by 1.0 years. This yields an “exchange rate” of 2 subjective years lost per 1 chronological age gained. Other features have their own exchange rates, which may be manipulated to accumulate “net profit” in both SubjAge and PsychoAge dimensions.

Other directional feature analysis methods may be more appropriate for navigating the psychological aging landscape since linear mixed effect models operate based on multiple assumptions and simplifications. More specifically, they treat all features independently and approximate the complex interrelations between PsychoAge and SubjAge that may be in place with a random intercept. Accumulated local effects or more sophisticated Shapley value analysis may handle the convoluted feature interrelations more efficiently.

To further validate PsychoAge and SubjAge we tested their prediction errors (delta) as all-cause mortality risk factors (Figure 6). SubjAge delta was proven to be a more powerful risk factor than PsychoAge. More specifically, the SubjAge delta beyond ±5 years was associated with roughly doubling or halving the mortality rate.

We also tested the 50 psychosocial markers of aging as risk factors. We identified significant mortality risks associated with certain factors among (i) health features (“Health compared to others your age”, “Rate current health”, “Shortness of breath while walking up a slight hill”), (ii) personality traits (“Conscientiousness personality trait”, “Agency personality trait”), (iii) psychological beliefs (“Live for today”, “Positive reappraisal”, “Lower aspirations”), (iv) well-being (“Satisfied with life at present”), and, (v) demographic factors (“Chronological age”) (Figure 7).

A problem frequently encountered even by psychologists is obtaining sufficiently detailed information about their patients while keeping the data collection process as short as possible to avoid survey fatigue. In this work, we propose a solution to this survey length-descriptiveness balance problem based on modern deep learning and biogerontological methods. The solution is a relatively short list of features that are both modifiable and provably important in the context of aging.

Some studies show that family history is a source of numerous highly important aging-related features [35]. For example, having long-lived parents and grandparents is strongly correlated with a longer lifespan. However, such factors are not easily modified, especially if the grandparents are no longer alive. Therefore, in this study, we have deliberately limited questions on non-modifiable historical factors to give our surveys more practical value. We demonstrated that variables related to health and closer personal relationships play a crucial role in chronological and subjective age prediction. Furthermore, images about life changes, for instance, when females or males enter middle age, demonstrate a strong predictive power. We suggest that modifying the behavior and the mindset via these variables may be a promising therapeutic concept.

The factors comprising the developed aging clocks can be used to create individual behavioral therapies that would make them feel and actually become biologically younger. For example, PsychoAge can be used to quantify the beneficial effects of daily vigorous-intensity activity on their rate of aging. SubjAge, in its turn, can be used to quantify the beneficial effects of physical activity on personal perception of age.

Focusing on such modifiable age-related features while being able to score lifestyle choices numerically offers interesting opportunities to both professional therapists and individuals seeking self-improvement. We believe that the described approach has a high potential to increase longevity conscience on a population level.

When the category of close relationships is considered, people with access to PsychoAge and SubjAge may choose to develop stronger bonds, get married, or stay married out of an egoistic incentive to prolong their healthspan. The beneficial effect of close relationships and intimacy on health was previously shown in multiple studies, and we believe that drawing people’s attention to such physical-mental health connections should not be neglected [36].

Extracting actionable items from human biological profiles, such as transcriptomic or proteomic profiles, is an actively researched subject. The profiles associated with human psychology can also be subjected to similar workflows to devise personal behavioral therapy plans. In this study, we have demonstrated how the combination of deep learning and aging clocks can be used to create psychological surveys that promote longevity consciousness and personal improvement. These tools and methods could be applied in a wide range of research areas, including psychiatry, longevity, psychology, and psychophysiology for the greater good of society.

## MATERIALS AND METHODS

In order to develop psychological clocks and to examine the relationship between both chronological and subjective age, and between key bio-, socio-, and psychological factors, a series of DNNs were trained based on data from anonymized questionnaires responses from U.S. residents that were acquired during the Midlife in the United States longitudinal survey (MIDUS 1, MIDUS 2, MIDUS Refresher, http://www.midus.wisc.edu/). Our aim was to choose psychological, social, and health factors that could be modified through the clinical intervention or participants’ behavioral change.

To specifically focus on modifiable factors, we eliminated questions from the MIDUS 1 survey that featured non-modifiable factors. These included questions related to chronological age, genetic predispositions, or past events (i.e., the age of death of family members, age of children), non-modifiable past experiences (i.e., childhood experiences), race, and birthplace.

In this article, we employ the term “chronological age”, when the age at the time of the phone interview is predicted based on psychological and behavioral parameters. We employ the term “subjective age”, when the age participants feel like most of the time is predicted. The exact questions asked to the participants were: (i) the age at the time of the phone interview (chronological age) and (ii) the age participants felt like most of the time (“Now imagine you could be any age. What age would you like to be?”).

### Data

The MIDUS series was funded by the John D. and Catherine T. MacArthur Foundation Research Network on Successful Midlife Development. MIDUS 1 data was collected between 1995 and 1996. The study consisted of two parts – In Part 1, the participants were asked to undertake a 30-minute telephone interview while Part 2 consisted of a self-administered questionnaire, which included sociodemographic characteristics, physical health, biomarkers, and psychosocial information, including the estimation of the subjective age. In total, the dataset contained surveys from a total of 7,108 participants.

In order to verify our findings, the MIDUS 2 (https://www.icpsr.umich.edu/web/NACDA/studies/4652, 2004-2006) and MIDUS Refresher datasets (https://www.icpsr.umich.edu/web/NACDA/studies/36532, 2011-2014) were used. MIDUS 2 (2002-2009) is a follow-up of the MIDUS 1 study (1995-1996), while the MIDUS Refresher (2011-2014) is a next-wave study with new participants. To examine the predictive power of trained models on the publicly available data, we analyzed the MIDUS 2 and Refresher datasets and tested our DNN on these datasets retrospectively.

To perform each analysis, MIDUS 1 was split into training and test datasets. The training cohort of MIDUS 1 consisted of 7,106 participants (3,176 females). Following preprocessing, the dataset consisted of 6,071 participants (2,523 females). The testing dataset consisted of 1,214 participants (644 females). An overview of the process is given in Figure 2.

The validation dataset – MIDUS 2 – consisted of participants 4,963 (2,647 females). After preprocessing, the dataset consisted of 2131 participants. The validation dataset MIDUS R consisted of participants 3,576 (2,647 females). After preprocessing, the dataset consisted of 2,521 participants (1,337 females).

For reporting purposes, all datasets were divided into three age-cohorts: 25-39, 40-64, 65-75 years, as per MIDUS 1 specifications.

To perform mortality analyses, we explored the MIDUS 2 and MIDUS Core Sample Mortality datasets (1,767 confirmed deaths: https://www.icpsr.umich.edu/web/NACDA/studies/37237, 2016).

### Feature selection and data preprocessing

The original MIDUS 1 dataset had a total of 2,097 defined features. To perform feature engineering and data preprocessing we excluded the following features: explanatory and ID features and highly age-related features (e.g. age group). Then, the samples with either target variable (subjective or chronological age) outside the 0-100 years range were excluded. Next, we calculated a feature correlation matrix and highly correlated (Pearson’s r > 0.9). We selected features that have the most predictive power, other correlated features were excluded. Features were then filtered to remove those that contained >80% missing values. All categorical features were binarized using one-hot encoding. Finally, outliers were filtered and removed using the Isolation Forest outlier detection algorithm.

After the filtering and exclusion processes, the final dataset contained 6,071 participants with a total of 954 features, of which 573 were defined as categorical and 382 as numerical.

### Training procedure

First, we trained a chronological age predictor on a full list of features remaining after feature engineering and data preprocessing (here, “features” were defined as those that imply preprocessed or raw input data). Then feature importance analysis was applied to select the 100 most important features (see “Methods: Feature importance analysis”). After another round of training 50 most important features were selected to build the final models: PsychoAge for chronological age prediction and SubjAge for subjective age prediction.

A complete list of the fifty features is provided in Supplementary Table 1. All features were divided into the following categories, according to their MIDUS description: (i) health, (ii) closer relationships, (iii) images about life changes, (iv) occupational features, (v) community involvement, (vi) personality traits, (vii) psychological beliefs, (viii) demographic features, and (ix) well-being.

**Figure 8:**
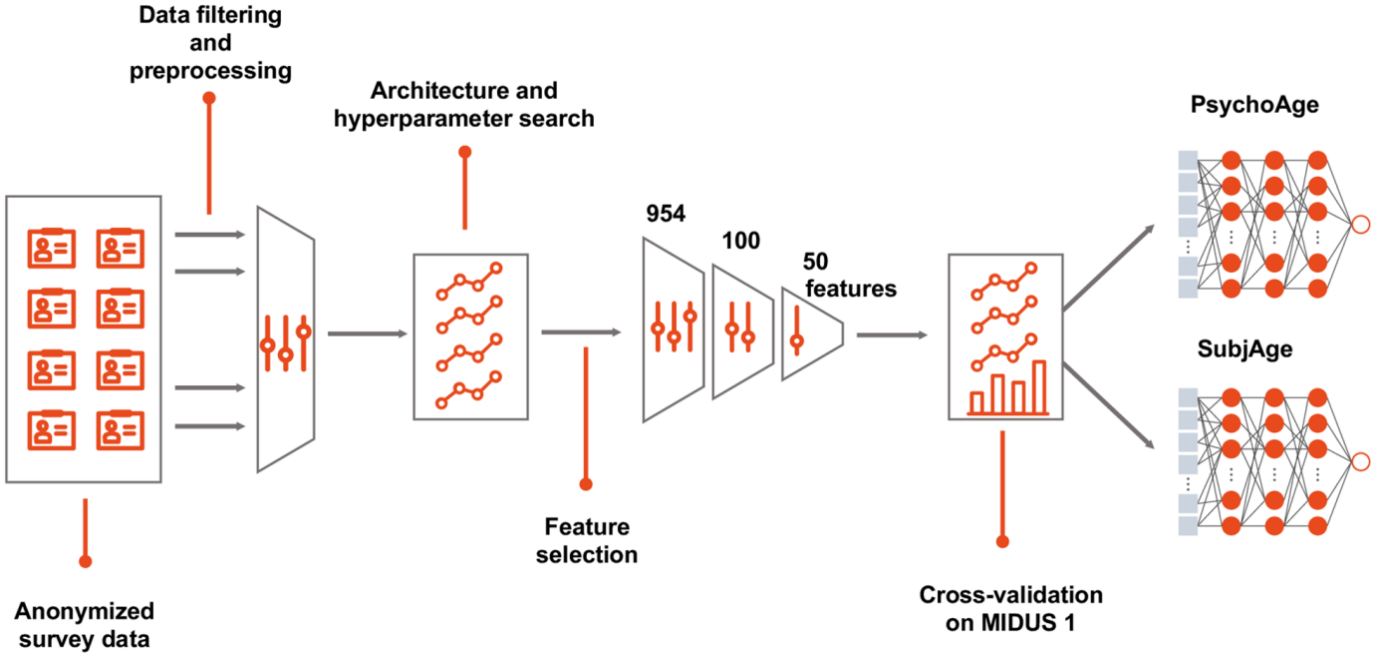
**Building a deep psychological age predictor.** MIDUS 1 dataset was filtered to contain only relevant features and samples with <20% missing values. DNNs were built with all the available features in a cross-validated manner to select the best hyperparameters. After establishing feature importance, top-100 features were selected, which were further filtered down to top-50 features. The selected top-50 features were used to build the final PsychoAge and SubjAge models.

Final models were trained with five-fold cross-validation (CV) using all MIDUS 1 samples. MIDUS 2 and MIDUS Refresher were used for model validation purposes (Figure 8). Final model performance was visualized using Seaborn for Python (v0.10.0; https://seaborn.pydata.org).

### DNN architectures

In this study age prediction was treated as a regression tasks, i.e., the model utilized vectors to calculate values for both psychological and subjective age estimation task. A single value was then returned for one of the selected age’s targets. Here we used a deep model composed of multiple layers to permit the fitting of data with high-level dependencies between the input features (biopsychological markers) and the output features (different types of ages).

Multilayered, feed-forward neural networks were used as deep models (i.e., models that had more than 3 hidden layers) with Python implementation using the Keras library (https://keras.io/) with Tensorflow backend (https://www.tensorflow.org) to build and train the neural networks. A grid search over the space of model parameters was applied to find the best performing network architecture. The MAE loss function was used as an objective for neural networks. The best performing neural network had 5 hidden layers with 512 neurons each. Mish activation non-linear pointwise functions were used to introduce non-linearity to the linear transformed input in each hidden layer of the neural network. Radam, with lookahead, was used to leverage a dynamic rectifier to adjust the adaptive momentum of Adam based on the variance applied to optimize the cost function. A dropout with 45% probability was used after each layer for regularization. L2 regularization was applied on the weights of all hidden layers with a coefficient equal to 0.0001. Neural networks were trained using five-fold cross-validation to compensate for overfitting and to obtain more robust performance metrics. All experiments were conducted using an NVIDIA RTX 2080Ti graphics processing unit.

### Feature importance analysis

To address the interpretability problem of DNNs, one model-agnostic (Permutation Feature Importance, PFI) and one DNN-specific (Deep Feature Selection, DFS) feature importance analysis method were used [37].

DFS was applied as a DNN-specific feature importance method. DFS was used to select features at the input level of a DNN. The primary role of DFS was to add a sparse one-to-one linear layer between the input layer and the first hidden layer of an MLP. To select input features, weights of the DFS layer were required to be sparse, to satisfy this requirement elastic net regularization was applied. DFS importance score was defined as the DFS weight of the input features.

PFI was applied as a model-agnostic feature importance method. PFI was applied to identify the best performing models for each dataset. PFI assigns an importance score to a feature by measuring the drop in model accuracy upon randomly permuting its values. A larger drop in model accuracy is associated with greater importance. To carry out PFI analysis on categorical features, which were encoded as one-hot columns, “1” was placed in a random column and other columns for the corresponding feature were turned to “0”.

The final importance score we report here is the mean of normalized PFI and DFS scores.

To identify which variables were most important for accurate prediction in specific age groups, a separate set of DNNs was trained using the predefined set of 50 features. Each of these DNNs was trained using only the samples that belong to one of the following age groups: 25-39, 40-64, 65-75 years (Table 2).

### Variable effect estimation

To interpret the available variables in terms of the effect they have on psychological aging, we employed an approach based on linear models with mixed effects.

All samples were assigned to one of the five PsychoAge or SubjAge groups (25-34, 35-44, 45-54, 55-64, 65-74 years predicted). For each variable assessed a random intercept for each age group was added to the model:

SubjAge~Variable+(1|PsychoAge group)

PsychoAge ~ Variable+(1|SubjAge group)

The mixed-effects analysis was carried out on the complete MIDUS 1 data set while using the predictions obtained in CV. The implementation was written in R 3.6.2, mixed-effects models were implemented with lme4 package (v1.1.21; https://cran.r-project.org/web/packages/lme4/index.html).

Visualization of effects was conducted with Plotly (v.4.5.0; https://plotly.com) for Python.

Model validation was carried out using MIDUS 2 and MIDUS Refresher datasets. This pipeline was repeated independently for PsychoAge and SubjAge.

### Survival analysis

To investigate the predictive ability of deep psychological aging clocks in terms of all-cause mortality, we employed Cox-regression models for both psychological age and subjective age. To evaluate the association of the predicted age with all-cause mortality, hazard ratios (HR) were calculated. Survival time data (defined as the age at examination until the age of death or last follow-up) was analyzed. For hazard analysis by group, the CoxPHFilter method was used from lifelines for Python (v.0.23.9; https://github.com/CamDavidsonPilon/lifelines). Cox models were adjusted for chronological age and sex.

For survival analysis purposes, the rate of aging was expressed as a set of one-hot binary variables representing the sample’s delta — the difference between predicted and the actual age of the samples (either chronological or subjective). One-hot columns were filled based on whether a sample’s delta was below -5 years, above +5 years, or within the ±5 year error range.

### Statistical analysis

The following metrics were used to evaluate the accuracy of the age prediction models:

1) Coefficient of determination: $R^{2}=1-\frac{\sum_{i=1}^{N}{\left({\hat{y}}_{i}-y_{i}\right)}^{2}}{\sum_{i=1}^{N}{\left(y_{i}-y^{\prime }\right)}^{2}};y_{i}$ is the real value, ${\hat{y}}_{i}$ is the predicted value and $\underline{y}$ is the mean of *y*. *R*^2^ shows the percentage of variance explained by the regression between predicted and actual value.
2) Mean absolute error: $\text{MAE=}\frac{1}{N}\sum_{i=1}^{N}\left|{\hat{y}}_{i}-y_{i}\right|;$ where ${\hat{y}}_{i}$ is a predicted age, *y_i_* is a real value and N is the total number of samples. MAE demonstrates average disagreement between predicted and actual target value.
3) $\varepsilon -accuracy=\frac{\sum_{i=1}^{N}1_{A\;}({\hat{y}}_{i})}{N}$ where A = [*y_i_* – *ε*; *y_i_* + *ε*] and ${\hat{y}}_{i}$ is a value predicted by the model, *y_i_* is a true value. For example, if the DNN model predicted 55 for the sample with the actual target value ranging from 50 to 60, then this sample would be considered as correctly classified if the case epsilon equals 5.

### Limitations and future investigation

To develop a methodology for psychological and subjective age prediction we trained our models using the MIDUS data set based on participants in the United States. Future investigations that use more recent, and other national studies should be used to enhance the accuracy of the models Psychological deep clocks may be dependent on the socio-cultural values in a society. For example, if a society tends to shy away from a topic like sexual health, that factor may not show up as important for that culture’s psychological deep clock. Knowing the commonalities across psychological deep clocks will further refine the most important factors.

As these psychological deep clocks are integrated into clinical practice, hospitals and insurance companies may find these tools valuable in offering cost-effective medical care in addition to being able to obtain more value for the same amount of financial investment. Additionally, participants will spend less time answering 200+ survey questions and instead be able to focus on answering the most highly-ranked 50 questions. Clinicians will then know what are the best treatment protocols rather than relying on a blanket prescription (Supplementary Figure 4).

## Supplementary Material

Supplementary Figures

Supplementary Tables
