# Supplementary material for: PsychoAge and SubjAge: development of deep markers of psychological and subjective age using artificial intelligence
Source: Aging (Albany NY). 2020 Dec 8;12(23):23548–77. doi: 10.18632/aging.202344 (PMC7762465; doi:10.18632/aging.202344)
Supplement: Supplementary Figures [file aging-12-202344-s001.pdf]

SUPPLEMENTARY FIGURES

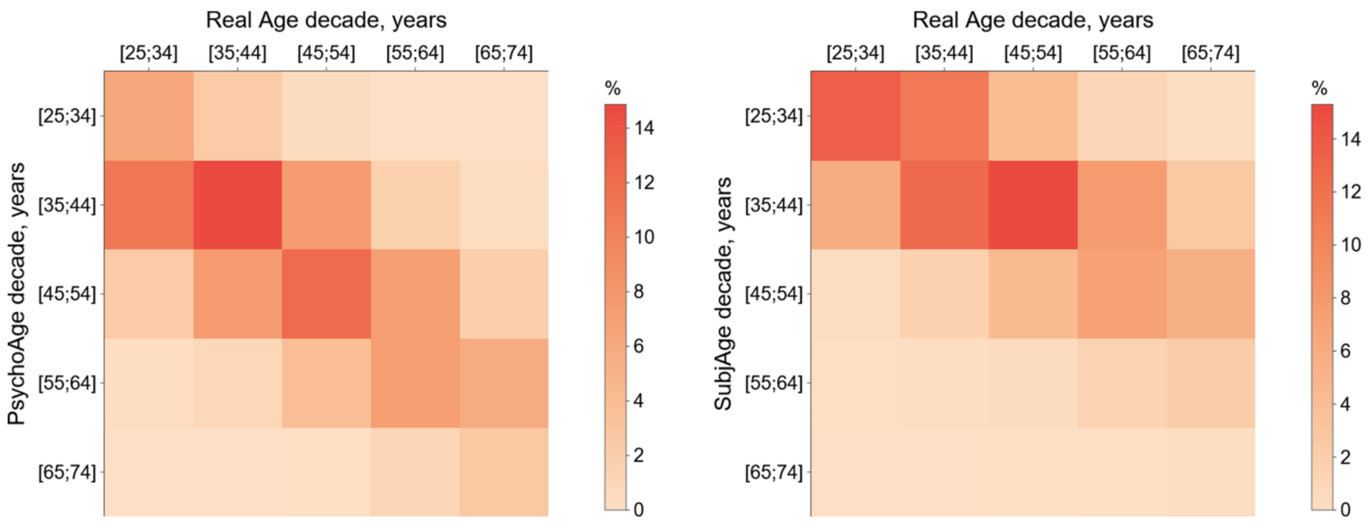

Supplementary Figure 1. Confusion matrix for PsychoAge and SubjAge divided by decades in MIDUS 1 (N=6071).

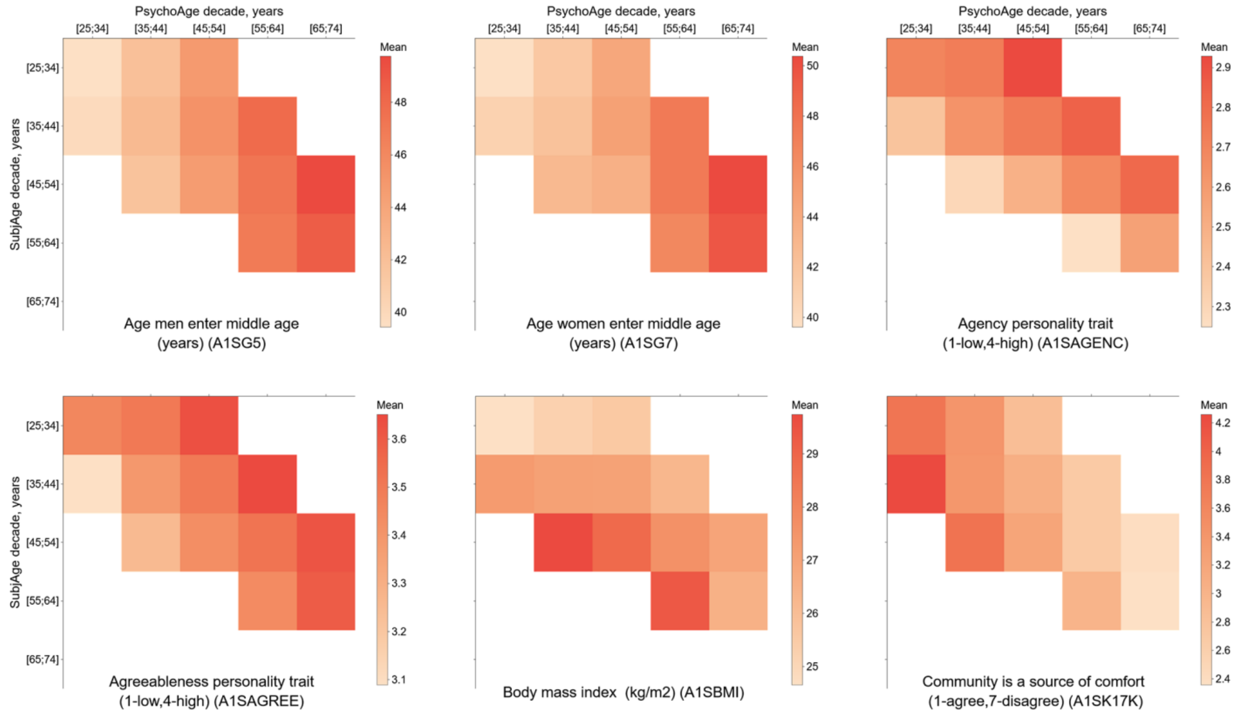

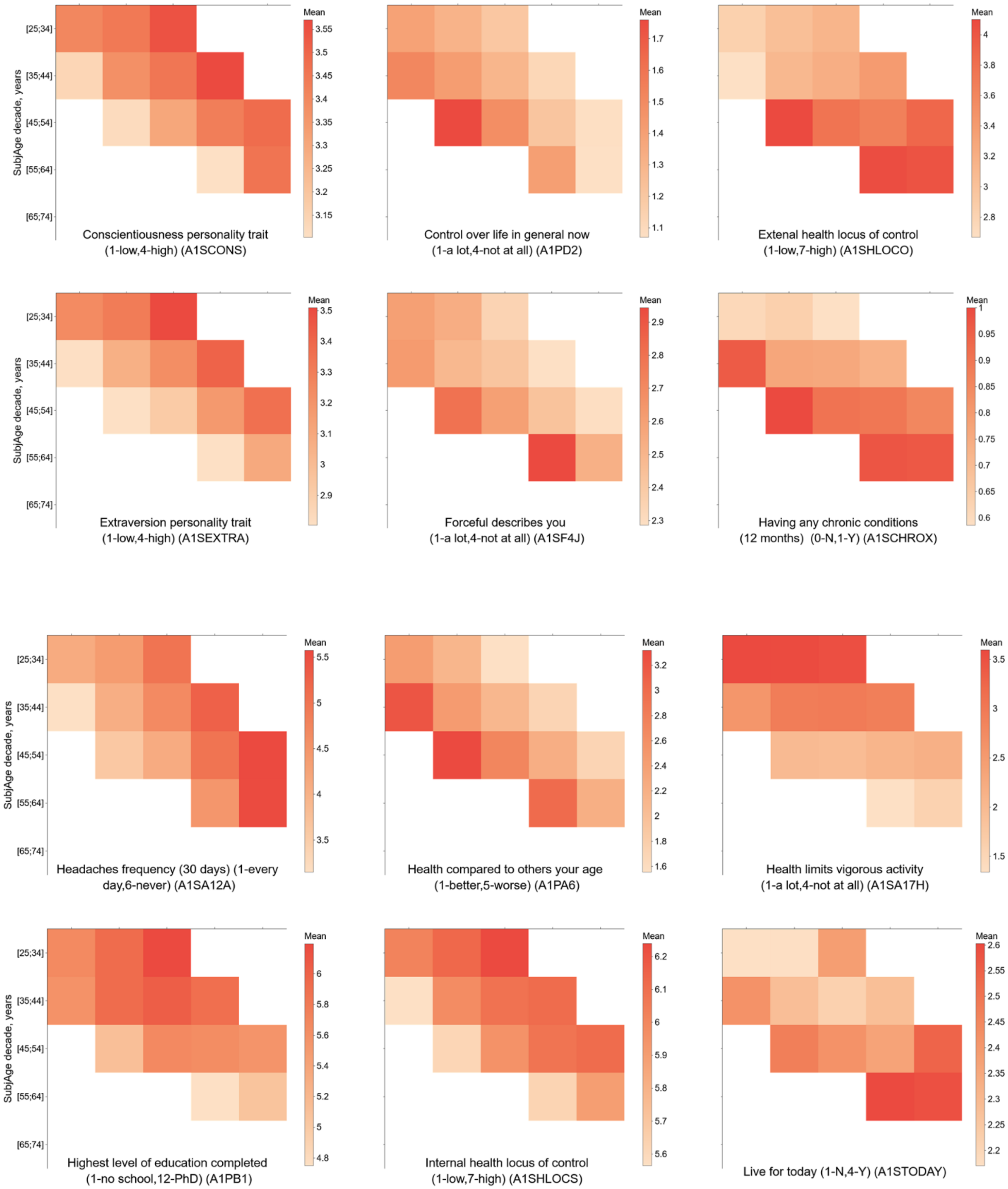
SubjAge decade, years
[25;34]
[35;44]
[45;54]
[55;64]
[65;74]
Mean
Conscientiousness personality trait (1-low,4-high) (A1SCONS)
Control over life in general now (1-a lot,4-not at all) (A1PD2)
Extenal health locus of control (1-low,7-high) (A1SHLOCO)
Extraversion personality trait (1-low,4-high) (A1SEXTRA)
Forceful describes you (1-a lot,4-not at all) (A1SF4J)
Having any chronic conditions (12 months) (0-N,1-Y) (A1SCHROX)
Headaches frequency (30 days) (1-every day,6-never) (A1SA12A)
Health compared to others your age (1-better,5-worse) (A1PA6)
Health limits vigorous activity (1-a lot,4-not at all) (A1SA17H)
Highest level of education completed (1-no school,12-PhD) (A1PB1)
Internal health locus of control (1-low,7-high) (A1SHLOCS)
Live for today (1-N,4-Y) (A1STODAY)

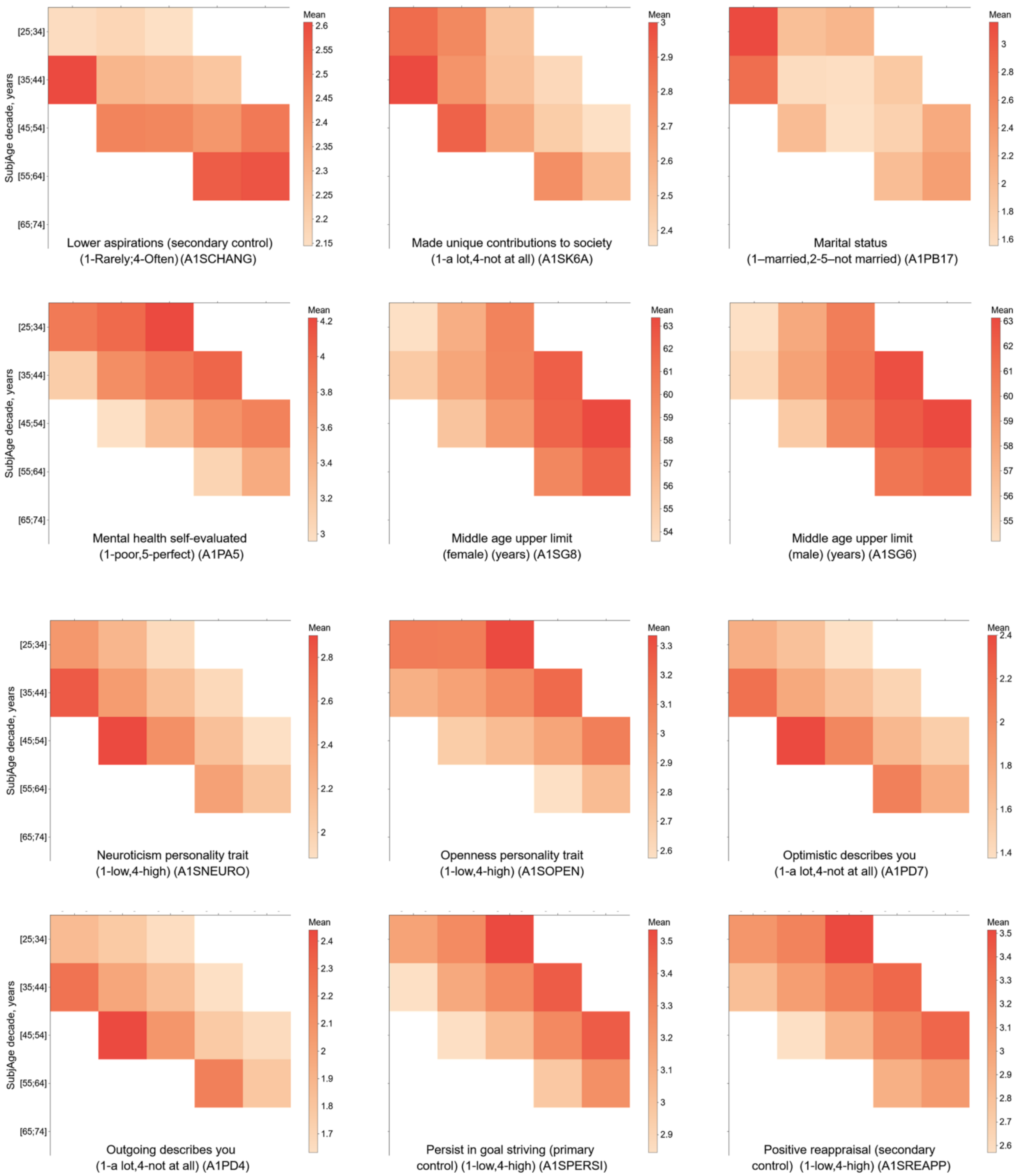

SubjAge decade, years
[25;34]
[35;44]
[45;54]
[55;64]
[65;74]
Mean
Lower aspirations (secondary control) (1-Rarely;4-Often) (A1SCHANG)
Made unique contributions to society (1-a lot,4-not at all) (A1SK6A)
Marital status (1–married,2-5–not married) (A1PB17)
Mental health self-evaluated (1-poor,5-perfect) (A1PA5)
Middle age upper limit (female) (years) (A1SG8)
Middle age upper limit (male) (years) (A1SG6)
Neuroticism personality trait (1-low,4-high) (A1SNEURO)
Openness personality trait (1-low,4-high) (A1SOPEN)
Optimistic describes you (1-a lot,4-not at all) (A1PD7)
Outgoing describes you (1-a lot,4-not at all) (A1PD4)
Persist in goal striving (primary control) (1-low,4-high) (A1SPERSI)
Positive reappraisal (secondary control) (1-low,4-high) (A1SREAPP)

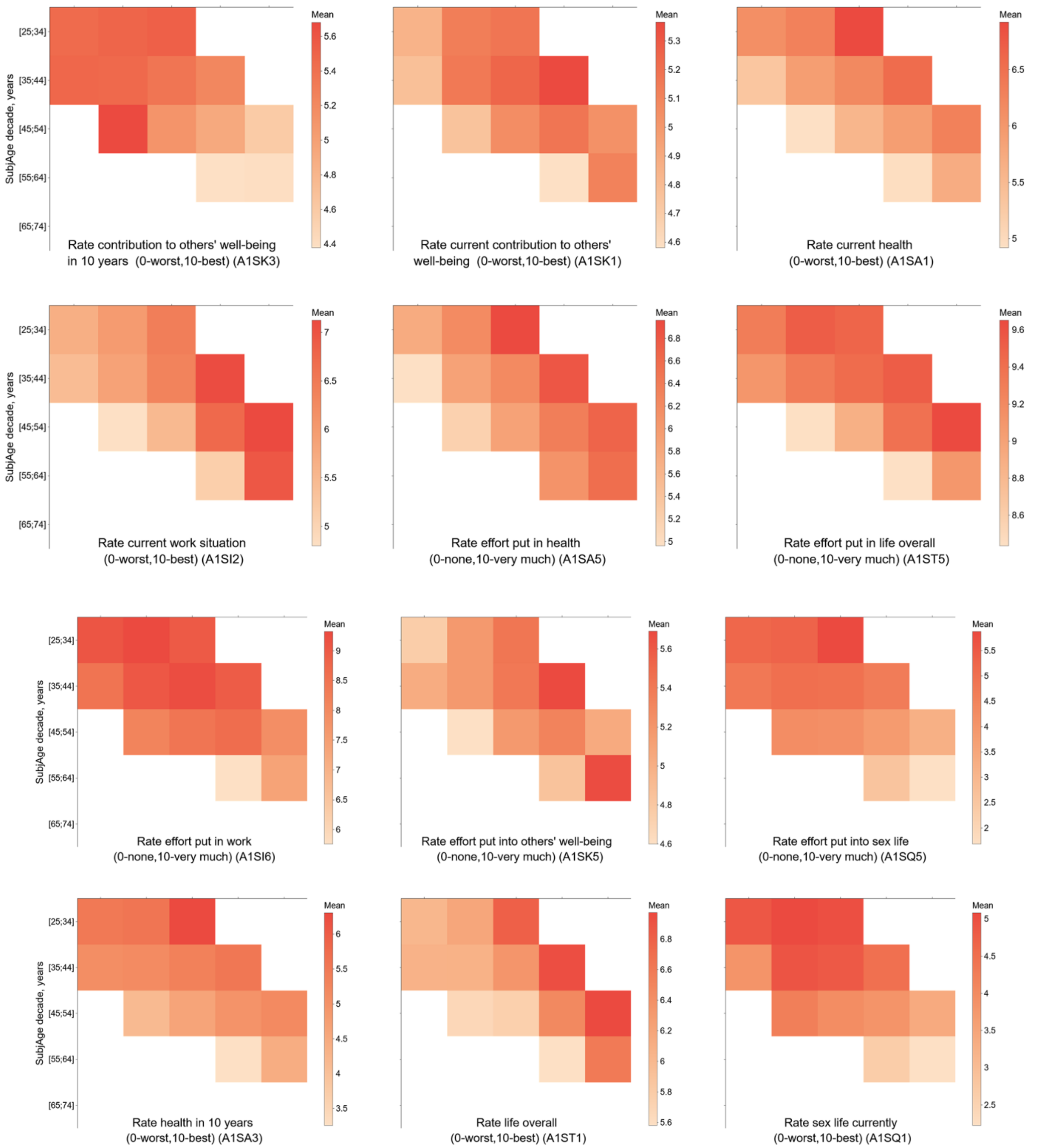
SubjAge decade, years
[25;34]
[35;44]
[45;54]
[55;64]
[65;74]
Mean
Rate contribution to others' well-being in 10 years (0-worst,10-best) (A1SK3)
Rate current contribution to others' well-being (0-worst,10-best) (A1SK1)
Rate current health (0-worst,10-best) (A1SA1)
Rate current work situation (0-worst,10-best) (A1SI2)
Rate effort put in health (0-none,10-very much) (A1SA5)
Rate effort put in life overall (0-none,10-very much) (A1ST5)
Rate effort put in work (0-none,10-very much) (A1SI6)
Rate effort put into others' well-being (0-none,10-very much) (A1SK5)
Rate effort put into sex life (0-none,10-very much) (A1SQ5)
Rate health in 10 years (0-worst,10-best) (A1SA3)
Rate life overall (0-worst,10-best) (A1ST1)
Rate sex life currently (0-worst,10-best) (A1SQ1)

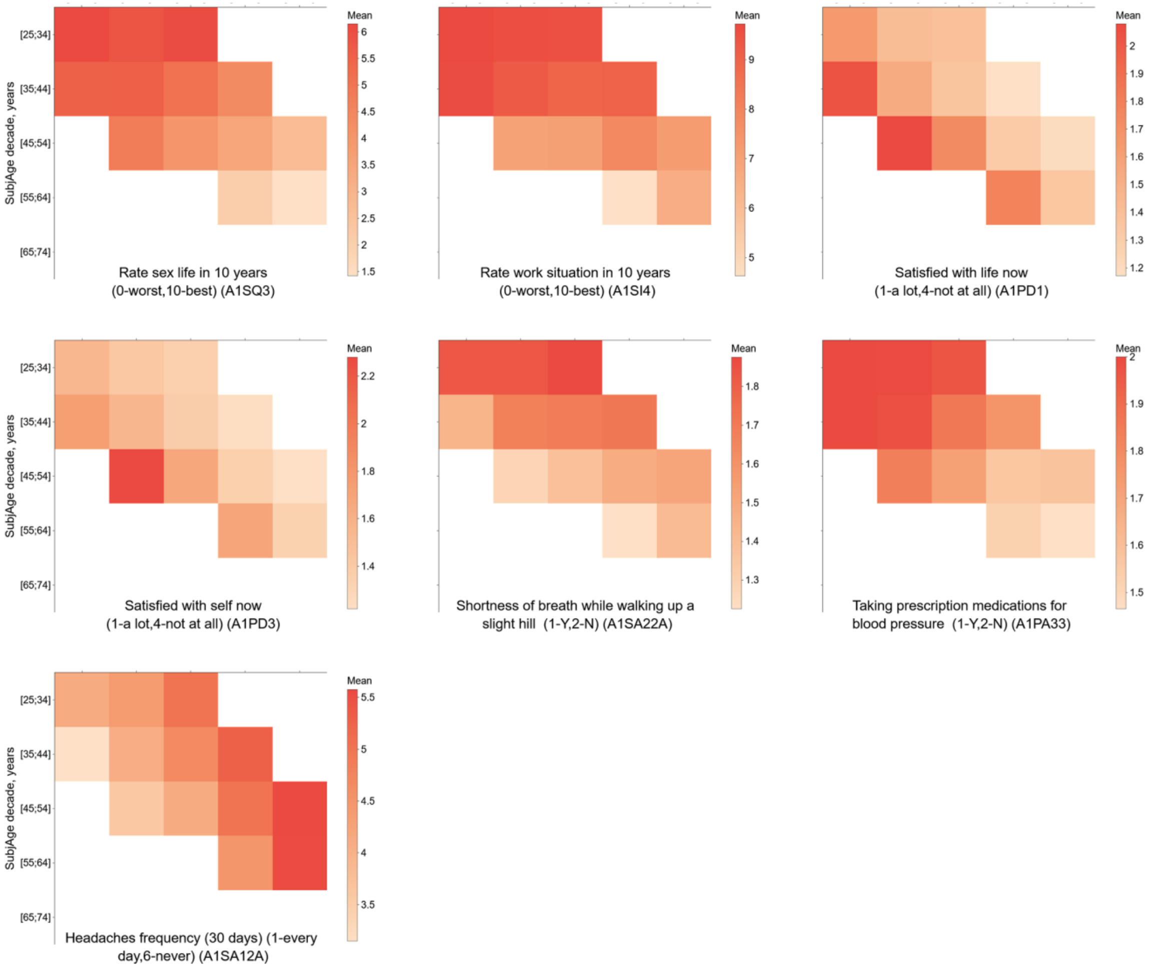

Supplementary Figure 2. Confusion matrices for all psychosocial variables used by PsychoAge and SubjAge, colored by mean variable value in the cross-prediction group. Squares with < 25 samples were left blank and excluded from mixed effects linear model analysis.

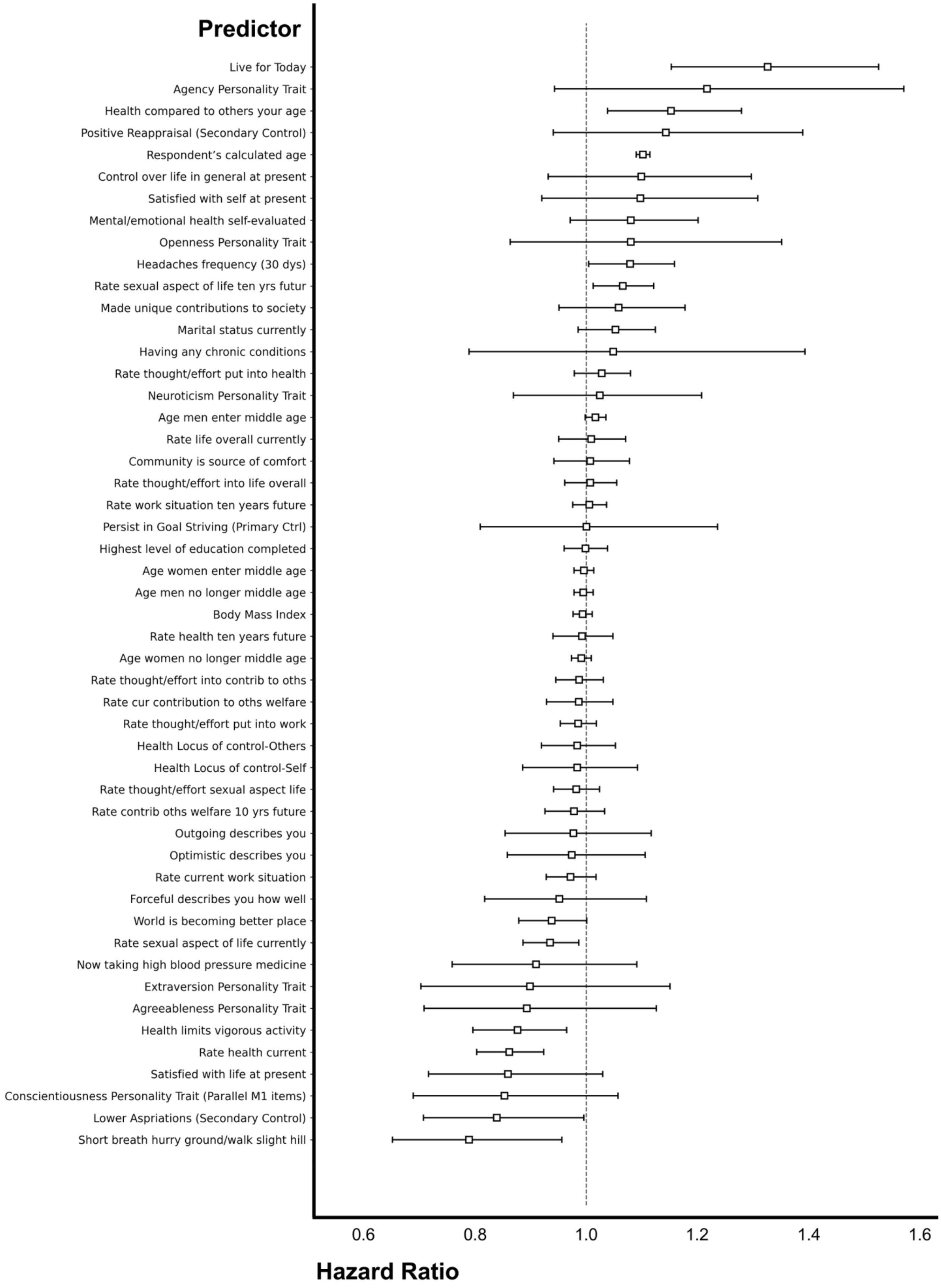

Supplementary Figure 3. The features that were associated with the greatest risk of mortality.

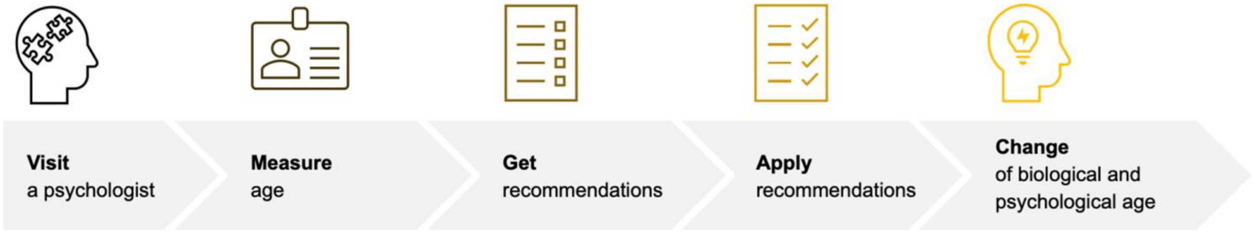

Supplementary Figure 4. Optimized patient flow with deep psychological clocks.
