# Supplementary material for: PsychoAge and SubjAge: development of deep markers of psychological and subjective age using artificial intelligence
Source: Aging (Albany NY). 2020 Dec 8;12(23):23548–77. doi: 10.18632/aging.202344 (PMC7762465; doi:10.18632/aging.202344)
Supplement: Supplementary Tables [file aging-12-202344-s002.pdf]

SUPPLEMENTARY TABLES

Supplementary Table 1. PFI importance scores for features used in PsychoAge.

Domains	Features	PFI value
**Health**	Health limits on vigorous activity	1.155
	Prescription medications for blood pressure	1.054
	Headaches frequency (30 days)	0.727
	Health compared to others your age	0.491
	Health locus of control - others	0.170
	Body mass index	0.135
	Mental/emotional health self-evaluated	0.100
	Thought/effort put in health	0.066
	Any chronic conditions (12 months)	0.061
	Shortness of breath during a little physical activity such as walking up a slight hill	0.033
	Health 10 years in future	0.027
	Health locus of control - Self	0.017
	Current opinion about health	< 0.01
**Closer relationships**	Rate sexual aspects of life 10 years in future	1.542
	Marital status	0.991
	Thought/effort sexual aspect of life	0.192
	Current opinion about sexual aspects of life	0.065
**Images of life changes**	Age men enter middle age	0.639
	Age women enter middle age	0.177
	Age men no longer identify as middle age	0.177
	Age women no longer identify as middle age	0.040
**Occupational features**	Current opinion about work situation 10 years future	0.498
	Current opinion about work situation	0.297
	Thought/effort in work	0.082
**Community involvement**	Contribution to others welfare 10 years future	0.400
	Community as source of comfort	0.275
	Unique contributions to society	0.092
	Current opinion about contribution to others' welfare	0.035
	World is becoming better place	0.033
	Thought/effort in contribution to others welfare	0.033
	Satisfied with self at present	< 0.01
**Personality traits**	Neuroticism as a personality trait	0.154
	Extraversion as a personality trait	0.070
	Openness as a personality trait	0.030
	Agreeableness as a personality trait	0.026
	Agency as a personality trait	0.020
	Conscientiousness as a personality trait	< 0.01
**Psychological Beliefs**	Forceful as a psychological trait	0.108
	Lower aspirations (secondary control)	0.089
	Live for today	0.003
	Positive reappraisal (secondary control)	0.028
	Persist in goal striving (primary control)	0.024
	Optimistic as a psychological trait	0.006
	Outgoing as a psychological trait	< 0.01

**Demographic features**	Highest level of education completed	0.129
	Sex	0.085
**Well-being**	Satisfied with life at present	0.091
	Current opinion about life overall currently	0.084
	Control over life in general at present	0.026
	Thought/effort in life overall	< 0.01

Supplementary Table 2. The performance of models trained on the MIDUS 1 dataset (all ages) and its age group subsamples (25-39, 40-64, 65-75).

Dataset	Chronolo-gical Age, years	R^2	MAE, years	Baseline MAE, years	ε-accuracy	N, people
**MIDUS 1**	25-39	0.07	3.35	3.56	1.0	2040
Psychological age	40-64	0.26	4.91	6.00	0.9	3339
	65-75	-0.09	2.45	2.39	1.0	682
	all ages	0.56	6.70	10.79	0.78	6071
Subjective age	25-39	0.01	5.23	5.04	0.88	2040
	40-64	0.15	6.89	7.40	0.76	3339
	65-75	0.05	7.63	8.07	0.72	682
	all ages	0.4	7.32	9.78	0.74	6071
**MIDUS 2**	25-39	-2.93	3.00	1.51	1.0	346
Psychological age	40-64	0.25	4.93	5.94	0.91	2492
	65-75	-0.07	2.52	2.70	1.0	738
	all ages	0.46	7.18	10.27	0.73	3870
Subjective age	25-39	0.05	4.90	4.87	0.9	346
	40-64	0.15	7.57	8.30	0.72	2492
	65-75	0.04	7.50	8.05	0.73	738
	all ages	0.32	8.53	10.72	0.66	3870
**MIDUS Refresher**	25-40	-0.02	2.97	2.86	1.0	591
Psychological age	40-65	0.21	5.56	6.40	0.88	1300
	65-75	-0.01	2.33	2.61	1.0	624
	all age	0.56	7.73	12.36	0.7	2521
Subjective age	25-40	0.07	5.67	5.62	0.85	591
	40-65	0.15	7.84	8.69	0.70	1300
	65-75	0.04	8.27	8.51	0.68	624
	all age	0.34	8.56	11.27	0.65	2521

DNNs were validated on two datasets: MIDUS 2 and MIDUS Refresher with 50 input features.
